# MicroRNA-146a switches microglial phenotypes to resist the pathological processes and cognitive degradation of Alzheimer's disease

**DOI:** 10.7150/thno.53418

**Published:** 2021-02-19

**Authors:** Chunmei Liang, Ting Zou, Miaoping Zhang, Weihao Fan, Tianzhen Zhang, Yuling Jiang, Yujie Cai, Feng Chen, Xiongjin Chen, Yuanhong Sun, Bin Zhao, Yan Wang, Lili Cui

**Affiliations:** 1 Guangdong Key Laboratory of Age-Related Cardiac and Cerebral Diseases, Institute of Neurology, Affiliated Hospital of Guangdong Medical University, Zhanjiang, China.; 2 Department of Neurology, Yuebei People's Hospital Affiliated to Shantou University, Shaoguan, China.; 3 Department of Pharmacology and Neuroscience, University of North Texas Health Science Center, Fort Worth, TX, USA.

**Keywords:** microRNA-146a, Alzheimer's disease, microglial polarization, neuroinfammation, phagocytic activity.

## Abstract

Alzheimer's disease (AD) is the most prevalent neurodegenerative disease and currently has no effective treatment. Mainstream research on the mechanisms and therapeutic targets of AD is focused on the two most important hallmarks, Aβ and Tau, but the results from clinical studies are not encouraging. Abnormal microglial polarization is a clear typical pathological feature in the progression of AD. Microglia can be neuroprotective by degrading and removing Aβ and Tau. However, under AD conditions, microglia transform into a pro-inflammatory phenotype that decreases the phagocytic activity of microglia, damages neurons and promotes the pathology of AD. We previously reported that a miR-146a polymorphism is associated with sporadic AD risk, and the nasal administration of miR-146a mimics reduced cognitive impairment and the main pathological features of AD. However, it is not clear by what mechanism miR-146a resists the pathological process of AD. In this study, we discovered that microglia-specific miR-146a overexpression reduced cognitive deficits in learning and memory, attenuated neuroinflammation, reduced Aβ levels, ameliorated plaque-associated neuritic pathology, and prevented neuronal loss in APP/PS1 transgenic mice. In addition, we found that miR-146a switched the microglial phenotype, reduced pro-inflammatory cytokines and enhanced phagocytic function to protect neurons *in vitro* and *in vivo*. Moreover, transcriptional analysis confirmed that miR-146a opposed the pathological process of AD mainly through neuroinflammation-related pathways. In summary, our results provide sufficient evidence for the mechanism by which miR-146a opposes AD and strengthen the conclusion that miR-146a is a promising target for AD and other microglia-related diseases.

## Introduction

Alzheimer's disease (AD) is the most prevalent neurodegenerative disease and results in progressive dementia. The two main features of this disease are intracellular neurofibrillary tangles (NFTs) composed of hyperphosphorylated tau (P-tau) and extracellular deposits of amyloid β (Aβ). Abnormal activation of microglia is a critical pathological process and is usually considered a downstream event caused by the deposition of Aβ in AD. As the resident immune effector cells in the brain, microglia are critically important for the homeostatic clearance of Aβ and for the modulation of synaptic formation, which is presumed to be involved in the pathogenesis of AD 1, 2. *In vivo* studies have shown that Aβ treatment activates microglia and exacerbates inflammatory responses by binding to innate immune receptors on microglia 3-5, and the hallmarks of Aβ plaques and NFTs are closely associated with microglial activation and neuroinflammation in AD pathology 6. Aβ clearance involves phagocytosis and endocytosis by microglia, and this type of microglia generally has a neuroprotective phenotype. In contrast, under AD conditions, the microglia switch to the neurotoxic (pro-inflammatory) phenotype during the advanced stage of AD, and pro-inflammatory cytokines also decrease the phagocytic activity of microglia, increase Aβ levels and exacerbate tau pathology, contributing to AD pathology 7, 8. Therefore, balancing or switching between microglia phenotypes may be a promising strategy for modulating the progression of AD.

We focused on the effects of non-coding RNA on different nerve cell functions in AD pathology and looked for appropriate therapeutic targets. MicroRNA-146a-5p (miR-146a), a highly conserved miRNA that is abundant in the nervous system, is well known for its important regulatory role in the immune response and inflammatory pathway 9, 10. As an NF-κB target gene, miR-146a directly targets a series of mRNAs encoded by inflammation-related genes such as complement factor H (CFH), interleukin (IL)-1 receptor-associated kinase 1 (IRAK1), and tumour necrosis factor (TNF) receptor-associated factor 6 (TRAF6), which is often regarded as a negative feedback regulator of the TLR4 pathway and ultimately plays a role in the neuroinflammatory response in the central nervous system (CNS) 11-13. Abnormal miR-146a levels in AD transgenic mice and human AD patients have been observed 14-16. We previously reported the association between miR-146a polymorphic loci and the risk of cognitive decline in AD patients 17 and further found that nasal administration of miR-146a mimics reduced cognitive impairment and the main pathological features of AD in a mouse model 18, suggesting that miR-146a has a critical role in AD and is a potential target involved in the occurrence and development of AD. However, it is not clear what mechanism of miR-146a opposes the pathological process of AD.

In this study, we aimed to further assess whether microglia-specific upregulation of miR-146a can improve the pathology of AD and to explore the underlying mechanism. Here, we report that microglia-specific miR-146a overexpression reduced learning and memory cognitive deficits and ameliorated plaque-associated neuritic pathology and neuronal loss in APP/PS1 transgenic mice (APP/PS1 Tg mice). Moreover, we reported that miR-146a switched microglial polarization to protect neurons under AD conditions *in vitro* and *in vivo*. The regulatory pathways and potential downstream targets of miR-146a involved in the amelioration of AD progression were also analysed in depth to provide evidence for the application of these potential targets in AD.

## Materials and Methods

### Animals

7-month-old wild-type (WT) C57BL/6J and B6/JNju-Tg (APP/PS1) male mice were obtained from GemPharmatech (Jiangsu, China) and housed in the SPF-grade Experimental Animal Center of Guangdong Medical University with a 12:12 h light:dark cycle and access to food and water. Each APP/PS1 mouse was kept in a cage alone. At the age of 10 months, all the mice were divided into four groups (WT-AAV-Mcon, WT-AAV-M146a, APP/PS1-AAV-Mcon, and APP/PS1-AAV-M146a), and the corresponding adenoviruses were injected with a stereotaxic apparatus.

### Stereotaxic surgery

Briefly, 10-month-old mice were anaesthetized with 5% chloral hydrate (0.7 mL/100 g body weight), hair was removed with depilatory paste, and the skin was cut with scissors before two small holes were drilled into the skull using a Dremel device attached to a stereotaxic frame. Next, according to the horizontal position of the skull, which is determined by bregma and lambda, the midline and depth stereotaxic coordinates of the brain were determined. Then, the mice received a bilateral injection of 1 μL microglia-specific miR-146a adeno-associated virus (AAV-M146a), which was expressed in a F4/80p-EGFP-MCS-SV40-PolyA vector, or blank vector (AAV-Mcon) (AP -2.3 mm, ML ± 2 mm, DV -1.8 mm; injected with 0.1 μL/ min). The needle was left in place for 5 min at the end of each injection. During the whole operation, the breathing of the mice was monitored to prevent asphyxia due to the prone position and the clamping of the stereotaxic apparatus. After stereotaxic surgery, the mice were monitored until full recovery from the anaesthesia and housed under standardized conditions for one month before behavioural tests.

### Morris water maze test

The Morris water maze test was conducted as previously described in detail 18. Mice from each group were placed in a cylindrical container with a diameter of 170 cm and a depth of 50 cm containing water at a temperature of 23-25 °C and allowed to swim freely for 90 s. Then, the Morris water maze test was formally carried out. The learning and memory abilities of mice were tested by positioning navigation experiments and space exploration experiments, respectively. The first day was the exposed platform adaptation period, and the swim speed and escape latency data are shown in Figure S1D-E. On the second day, the platform was submerged in the water, and the mice could not see it. During the five days of the positioning navigation experiment, we gently put the mice into the water at the four water entry points facing the wall of the pool and measured the time it took for the mice to find and climb on top of the platform within 90 s, which is called the “escape latency”. On the sixth day, we removed the platform and recorded the target quadrant residence time, the number of platform area crossings, and the target quadrant distance percentage for the space exploration experiment. All trials were videotaped and analysed by Morris software in a double-blind manner.

### Novel object recognition test

On the first day, each animal was placed in a 50x50 cm arena with no objects to explore for 10 min. Two identical objects were placed on opposing sides of the arena on the next day, with the mouse facing the two objects at the same distance from the tip of the nose. Following 10 min of investigation, the animals were removed and returned to their home cages. On the third day, one object was replaced with a novel object, and the animals were allowed 10 min of investigation. Contact with the two objects was recorded, including the number of times the nose or mouth touched the object and the time exploring within 2-3 cm of the object. All trials were videotaped and analysed by SuperMaze software in a double-blind manner.

### Immunohistochemistry

After the behavioural tests, the mice were anaesthetized and transcardially perfused with saline. Then, the brains were removed, and the hemispheres were separated. Brain samples assigned for immunohistochemical staining were immersed in OCT at -80 °C before coronal sectioning on a Frozen slicer (Thermo Fisher Scientific, USA) (10 μm thickness). The sections were restored to room temperature, fixed with cold acetone for 5 min, and permeabilizing agent was used for 5 min. Then, the cells were rinsed three times at room temperature for 5 min each with 1x PBS (pH 7.2-7.4) and blocked with 10% bovine serum in 0.1% Triton X-100 for 30 min. The blocking solution covered all brain tissues and prevented the tissue from drying out. Then, the samples were incubated with primary antibodies overnight at 4 °C. The primary antibodies included rabbit anti-NeuN (1:500, Abcam), rabbit anti-GFAP (1:200, CST), rabbit anti-Iba-1 (1:100, Abcam), rabbit anti-CD68 (1:300, Abcam), rabbit anti-APP (1:100, Millipore), and mouse anti-β-amyloid specific for Aβ_42_ (1:200, CST). The next morning, the sections were incubated for 1 h at room temperature with DyLight 488-/555-conjugated goat anti-rabbit/mouse IgG (1:200, Abcam) and stained with DAPI for 3 min. Fluorescence images were captured using an Olympus FV3000 confocal microscope.

### Thioflavine S staining

Tissue sections (20 μm) were fixed with cold acetone for 5 min, rinsed three times for 5 min each with 1x PBS (pH 7.2-7.4) and blocked with 10% bovine serum in 0.1% Triton X-100 for 30 min. The tissue sections were then free-floated in 20 mL of 1% thioflavin S (Thios) (Sigma, St. Louis, MO) aqueous solution for 5 min, followed by differentiation in 50% ethanol three times for 5 min each and washing in 1x PBS for 5 min. The sections were mounted with anti-fluorescence quenching sealing liquid. Images (4x magnification) were taken on the EVOS FL Auto Cell Imaging System and analysed using ImageJ. All images were preprocessed using the same threshold setting prior to analysis.

### TUNEL staining

Tissue sections (20 μm) were fixed for 30 min with 4% paraformaldehyde and washed twice for 10 min each with 1x PBS. Then, the sections were incubated at room temperature for 5 min with immunostaining strong penetrating liquid and washed twice with 1x PBS. Next, 50 μL TUNEL detection solution was added to the samples and incubated for 60 min at 37 °C. Finally, after rinsing three times with 1x PBS, we mounted the sections, observed the samples under an Olympus FV3000 confocal microscope and analysed the data using ImageJ. The excitation wavelength of Cy3 is 550 nm, and the emission wavelength is 570 nm, according to the One Step TUNEL Apoptosis Assay Kit (Beyotime, China).

### Extraction of microglia from adult mouse brain tissue

The dissociation of neural cells from adult mouse brain tissue free of debris and red blood cells was performed according to the Adult Brain Dissociation Kit instructions (Miltenyi Biotec, Germany). In brief, enzymes P, Z, Y and A were used to enzymatically digest brain tissue in a gentleMACS C tube that was attached upside down to the sleeve of a gentleMACS Octo Dissociator with Heaters running the 37 °C-ABDK-01 program. After termination of the program, the digested tissue was applied to a MACS SmartStrainer (70 µM) placed on a 50 mL tube, and the cell suspension was centrifuged at 300 g for 10 min at 4 °C. Then, debris and red blood cell removal was performed to harvest the neural cell suspension. Finally, the neural cell suspension was incubated with FcR Blocking Reagent to prevent antibodies from binding to Fc receptors and then labelled with CD11b-APC antibody (Miltenyi Biotec, Germany) for 10 min in the dark in a refrigerator at 4 °C to mark the microglia. To determine compensation settings, we used the isotype-matched control antibody as the negative control. Microglia-bound antibodies were detected with a FACSAria II Cell Sorter (BD Biosciences, USA) and harvested for the subsequent detection of miR-146a expression.

### ELISA quantification analysis

Cerebral tissue was homogenized in 1× RIPA buffer, incubated on ice, and centrifuged for 20 min at 12000 rpm. The supernatants were transferred to a new tube for IL-1β, TNFα, IL-6, and soluble Aβ_42_ and Aβ_40_ detection. Then, the precipitate was dissolved in 100 µL formic acid and 1900 µL Tris, and insoluble Aβ_42_ and Aβ_40_ were detected. Protein levels were determined by using a BCA assay (Thermo Fisher Scientific, USA) according to the manufacturer's instructions. The levels of soluble and insoluble Aβ_42_ and Aβ_40_ were determined using Aβ_42_ or Aβ_40_ ELISA kits (IBL, Japan). Similarly, the levels of IL-1β, TNFα, and IL-6 were determined using IL-1β, TNFα, or IL-6 ELISA kits (GenStar, China). All ELISAs were performed according to the manufacturers' instructions. Briefly, the tissue homogenates were added to a 96-well ELISA plate and reacted with primary and secondary antibodies. Then, 3,30,5,50-tetramethylbenzidine (TMB) was used as the substrate, and the results were quantified using an Epoch Microplate Spectrophotometer (BioTek, USA) at 450 nm. Averages from three replicate wells were used for each sample. All quantitative analyses were performed by the external standard method, producing a standard curve with a correlation coefficient >0.99.

### Chemical compounds

The “miR-146a mimic NC” and “miR-146a inhibitor NC” groups were scrambled sequences with no significant homologous sequence with the genome. The miR-146a mimics represent sequences of the mature form of miR-146a synthesized *in vitro*. The miR-146a inhibitor sequence was also directly chemically synthesized and was the complementary strand of the miRNA mature form with a 2'-methoxy modification. Its inhibition mechanism is mainly competitive binding to inhibit the function of the miRNA.

### Cell culture and transfection

Human microglial (HMC3) cells were purchased from Shanghai Zhong Qiao Xin Zhou Biotechnology Co., Ltd. Human neuroblastoma (SH-SY5Y) cells were obtained from Shanghai GeneChem. Co., Ltd. These cells were plated in 6- or 12-well plates (Nest, China) and cultured in DMEM/F12 or high glucose DMEM (HyClone, USA) supplemented with 10% foetal bovine serum (FBS; Gibco, USA), 100 U/mL penicillin, and 100 mg/mL streptomycin (HyClone, USA). HMC3 cells transfected with miR-146a mimics/mimic NC) or miR-146a inhibitor/inhibitor NC (10 nM mimic or 100 nM inhibitor final concentrations) (RiboBio, China). After 24 h of transfection, the culture medium was removed and replaced fresh medium with 100 ng/mL LPS or 5 μM Aβ_42_ and cultured for 72 h or 12 h. Then, the HMC3 cells were either subjected to total RNA extraction for qRT-PCR, lysed to obtain protein samples for Western blot analysis or ELISA, or co-cultured with SH-SY5Y cells. For the co-culture assay, we used 1 µL CellTrace Far Red (Invitrogen, USA) in 1000 µL PBS to stain HMC3 cells, incubated them without light for 20 min, and then terminated the reaction with complete medium. Then, SH-SY5Y cells were co-cultured for 24 h, and SH-SY5Y cell apoptosis was analysed by flow cytometry at 630/661 nm according to the Annexin V-PE/7-AAD Kit (Yeasen, China). All cells were cultured in a humidified incubator at 5% CO_2_ and 37 °C.

### Immunofluorescence

Cells were incubated in confocal petri dishes with microbeads activated one hour prior and cultured for 3 h. After washing with 1× PBS three times, the cells were fixed in 4% paraformaldehyde for 15 min, incubated with permeabilization agent for 5 min, and blocked with 10% goat serum for 30 min at room temperature. After being covered with anti-α-tubulin antibody (1:200) in 5% BSA overnight at 4 °C, the cells were incubated with Alexa Fluor secondary antibody (goat-anti-rabbit 488, Abcam, 1:500) for 1 h at room temperature. Fluorescence images were acquired by laser scanning confocal microscopy (Olympus FV3000). For each group, data were collected from 10 images or 25 cells for statistical analysis.

### qRT-PCR

Total RNA was extracted from mouse brains or cells by using TRIzol (Invitrogen, USA), and equal amounts of total RNA were treated with Dnase I, followed by reverse transcription using the RevertAid First Strand cDNA Synthesis Kit (Thermo Fisher Scientific, USA) following the manufacturer's instructions. qRT-PCR was performed by using the SYBR Green method on a LightCycler 480 sequence detector system (Roche, Germany). Each cDNA sample was tested with three replicates. The transcription level of U6 or GAPDH was used as an internal control. The specific primers are listed in Table S1, and the target sequences of Nkd2 siRNA are listed in Table S3. The relative expression levels were calculated using the 2^-△△CT^ method.

### Bioinformatic analysis of transcriptome

Cerebral samples from the APP/PS1-AAV-Mcon and APP/PS1-AAV-M146a groups stored in liquid nitrogen were sent to Shengyin Biotech. (Shanghai, China), and total RNA was extracted for RNA sequencing. Gene co-expression networks were made and self-origanizing maps (SOM) clustering was performed using mouse neuroinflammatory genes (Table S2). Subsequently, a highly correlated significant relationship pair was selected to retain the value of correlation coefficient >=0.7 and p<0.01 to visualize the correlation network of gene co-expression in Cytoscape. Using the SOM clustering method, the genes in different groups were clustered according to expression value, and the high-expression clustering modules were identified. Moreover, the differentially expressed genes (with P-value < 0.05 and |log2FoldChange| > 0.58) between APP/PS1-AAV-Mcon and APP/PS1-AAV-M146a were analysed after RNA sequencing. Then, the differentially expressed genes were used to perform GO gene ontology enrichment analysis using the Gene Ontology website (http://geneontology.org/) and pathway enrichment analysis using the KEGG database (https://www.kegg.jp/). Furthermore, TargetScan software was used to predict the direct targets of miR-146a among the downregulated genes.

### Dual-Luciferase assays

293T cells were cultured in high glucose DMEM (HyClone, USA) supplemented with 10% FBS and 100 U/ml penicillin/streptomycin in a humidified incubator at 5% CO_2_ and 37 °C. The 3'UTR sequences of FoxO6, Nkd2, Cd4, and Stpg1, which were cloned into the psiCheck2 vector containing firefly luciferase and Renilla luciferase using XhoI and NotI, were obtained from GuangZhou IGE Biotech. Briefly, 293T cells were transfected with miR-146a mimics or mimic NC (100 nM final concentration) and each gene construct using Lipofectamine 3000 (Invitrogen, USA). After 48 h of transfection, cell lysates were processed for dual-luciferase assays using the Dual-Lumi™ II Luciferase Reporter Gene Assay Kit (Beyotime, China) and detected with a Mithras LB 940 multilabel reader (Berthold Technologies, Germany).

### Statistical analysis

All graphs show the means ± S.E.M. from at least three separate experiments. Statistical analysis was performed with GraphPad Prism software version 7.0, applying one- or two-way ANOVA, using the Geisser-Greenhouse correction, followed by post hoc Tukey's test to determine the p-values for multiple comparisons or a two-tailed unpaired t test with Mann-Whitney test to determine the p-values for two-group comparisons. The GO enrichment analysis was performed according to Fisher's exact test followed by a correction for multiple testing. P-values < 0.05 were considered to be significant.

## Results

### Microglia-specific miR-146a overexpression improves learning and memory cognitive deficits in APP/PS1 Tg mice

**Figure 1:**
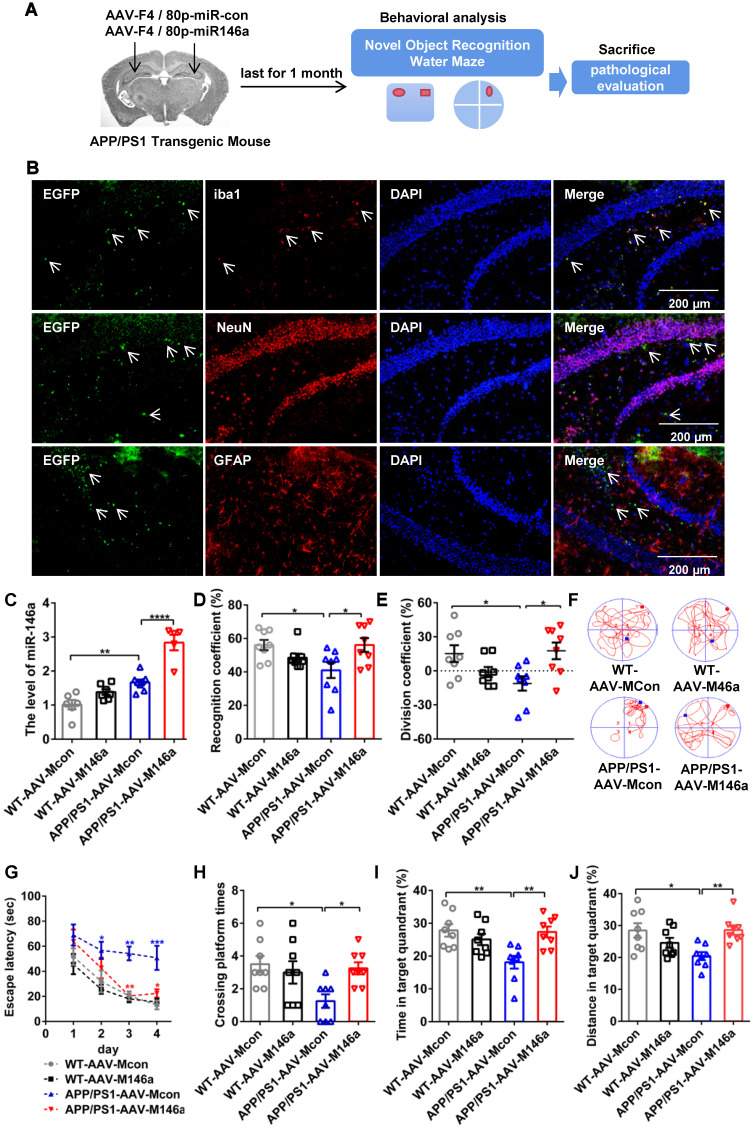
** Microglia-specific miR-146a overexpression improved cognitive deficits in learning and memory in APP/PS1 Tg mice.** A: Workflow for mouse behavioural analysis. B: Representative images of Iba1 (red), NeuN (red), and GFAP (red) surrounding microglia (green) and DAPI (blue) in the hippocampus of APP/PS1-AAV-Mcon mice and APP/PS1-AAV-M146a mice. Objective magnification: 20X, scale bar: 200 μm. C: qRT-PCR quantification of miR-146a-5p expression in microglia of each group (n=6). Statistical analysis was performed using one-way ANOVA followed by post hoc Tukey's test for multiple comparisons. D-E: Quantification of the recognition coefficient (D) and division coefficient (E) in the novel object recognition test of APP/PS1-AAV-M146a mice and APP/PS1-AAV-Mcon mice. One-way ANOVA with Tukey's multiple comparisons test. F: Motion tracking of mice in each group. G: The escape latencies of each group were tested in the Morris water maze for 5 consecutive days. Statistical analysis was performed using two-way ANOVA followed by post hoc Tukey's test for multiple comparisons (Interaction: Time*Treatment, P = 0.6357, no significant effect of two factors). H-J: Probe trials were performed at day 6, and the number of times crossing the platform site (H), the time spent in the target quadrant (I), and the swimming distance in the target quadrant (J) are shown. Statistical analysis was performed using one-way ANOVA followed by post hoc Tukey's test for multiple comparisons (H-J). For each group, n = 8. Data are the means ± S.E.M. *P<0.05, **P<0.01, ***P<0.005, and ****P<0.001.

Adeno-associated virus microglial miR-146a (AAV-M146a) was injected into the bilateral hippocampus of 10-month-old APP/PS1 Tg mice via the stereotaxic technique to evaluate the potential therapeutic effects on AD (Figure 1A). One month after administrating of AAV-M146a to APP/PS1 Tg mice (the APP/PS1-AAV-M146a group), we evaluated whether miR-146a was specifically expressed in microglia, as expected. The immunofluorescence results showed that exogenous miR-146a co-localized with Iba1 in the hippocampus of the APP/PS1-AAV-M146a group but did not co-localize with NeuN or GFAP (Figure 1B), suggesting that exogenous miR-146a was specifically expressed in microglia and not in neurons or astrocytes. Then, the levels of mature miR-146a, pre-miR-146a and miR-146a-3p were further evaluated, as shown in Figure S1. Compared with the AAV-Mcon group, higher levels of miR-146a and pre-miR-146a were observed in the AAV-M146a group (Figure S1A-B). However, no significant change in the miR-146a-3p level was observed (Figure S1C). Furthermore, we evaluated the expression level of miR-146a in microglia (Figure 1C). It is worth noting that the level of mature miR-146a in the brains of the APP/PS1 mouse group was higher than that in the brains of the wild-type mouse group, which was consistent with a previous study, suggesting that the level of miR-146a was elevated in the AD mouse model.

Then, the impact of AAV-M146a on cognitive impairment in the AD mouse model was evaluated by the Morris water maze (MWM) test and object recognition test (ORT). In the ORT, the APP/PS1-AAV-M146a group had better recognition behaviour, a higher recognition coefficient, and a higher division coefficient than the APP/PS1-AAV-Mcon group (Figure 1D-E and Figure S1L-M). In addition, consistent results were observed in the MWM evaluation, the APP/PS1-AAV-M146a group displayed significantly increased motion tracks and reduced escape latencies compared with the APP/PS1-AAV-Mcon group (Figure 1F-G and Figure S1F), but the swim speed and escape latency on the first day showed no different between the APP/PS1-AAV-Mcon group and APP/PS1-AAV-M146a group (Figure S1D-E and Figure S1J-K). In the spatial probe test, the APP/PS1-AAV-M146a group crossed the platform more often and spent more time, and had a greater swimming distance in the target quadrant than the APP/PS1-AAV-Mcon group (Figure 1H-J and Figure S1G-I). Collectively, these results suggested that microglia-specific miR-146a invention attenuated learning and memory cognitive deficits in this AD mouse model.

### Microglia-specific miR-146a overexpression attenuates neuroinflammation and switches microglial polarization in APP/PS1 Tg mice

**Figure 2:**
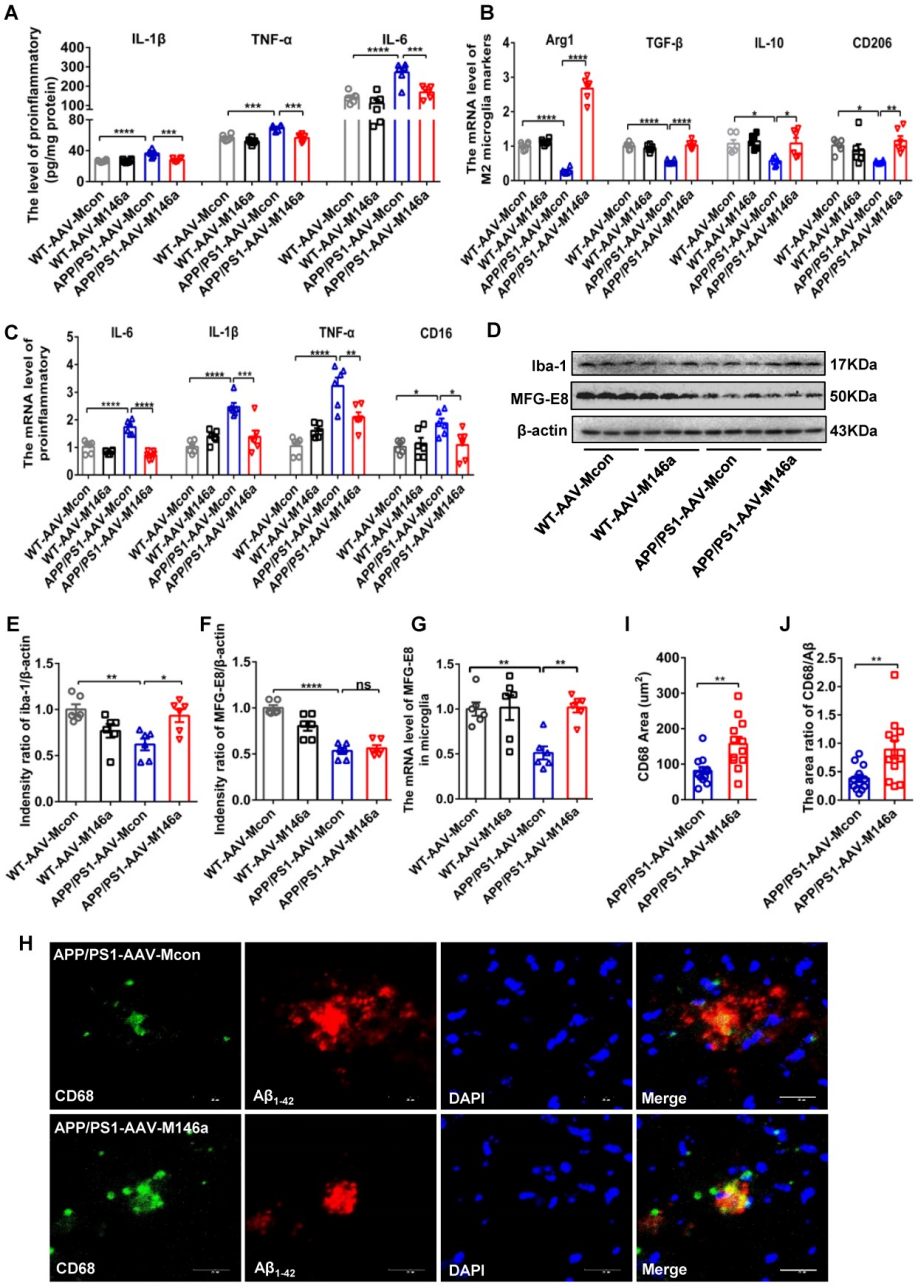
** Microglia-specific miR-146a overexpression attenuated neuroinflammation and microglial polarization in APP/PS1 mice.** A: The levels of cytokines (IL-6, IL-1β, and TNF-α) were analysed by ELISA with cerebral samples from each group (n=6). Statistical analysis was performed using two-way ANOVA followed by post hoc Tukey's test for multiple comparisons. B-C: Quantification of M2 phenotype microglial markers (Arg1, TGF-β, IL10 and CD206) and M1 phenotype microglia-secreted pro-inflammatory factors (IL-1β, IL-6, TNFα and CD16) in APP/PS1 AAV-M146a mice and APP/PS1-AAV-Mcon mice (n=6). Statistical analysis was performed using two-way ANOVA followed by post hoc Tukey's test for multiple comparisons. D: Total protein was extracted from cerebral tissue and separated by SDS-PAGE, and immunoblotting was performed with anti-Iba1, anti-MFG-E8 and anti-actin antibodies. E-F: Densitometry analysis was performed to quantify Iba1 and MFG-E8 levels in each sample, followed by normalization to actin loading control. Statistical analysis was performed using one-way ANOVA followed by post hoc Tukey's test for multiple comparisons. G: The mRNA levels of MFG-E8 in microglia was analysed by qRT-PCR of each group (n=6). Statistical analysis was performed using two-way ANOVA followed by post hoc Tukey's test for multiple comparisons. H: Representative images of CD68-immunolabelled activated microglia (green) surrounding plaques co-labelled for Aβ_42_ (red) in the hippocampus of AD mice and AD miR-146a mice. I: The area of CD68 was quantified in the hippocampus of APP/PS1-AAV-M146a mice and APP/PS1-AAV-Mcon mice. J: The area ratio of CD68/Aβ was quantified in the hippocampus of APP/PS1-AAV-M146a mice and APP/PS1-AAV-Mcon mice. Two-tailed unpaired t test with Mann-Whitney test were performed for I and J. Objective magnification: 20X, zoom: X6, scale bar: 20 μm. Data are the means ± S.E.M. and were analysed by one-way ANOVA with Tukey's test. *P<0.05, **P<0.01, ***P<0.005, and ****P<0.001; ns: no significance.

As the neuroimmune response of microglia, neuroinflammatory processes are critical in the development and progression of AD 19. To identify the potential role of microglial miR-146a in neuroinflammation under AD conditions, we analysed the typical inflammatory cytokines in APP/PS1 Tg mice after microglia-specific miR-146a overexpression. The results showed that miR-146a overexpression reduced the expression levels of inflammatory factors such as IL-1β, TNF-α, and IL-6 (Figure 2A). In addition, the downregulation of NLRP, ASC, and caspase-1 levels, which is indicative of NLRP3 inflammasome activation, was also inhibited by miR-146a overexpression in APP/PS1 Tg mice (Figure S1N). To further determine the effect of miR-146a on microglia in AD pathology, we evaluated the phenotype and polarization of microglia in APP/PS1 Tg mice administered AAV-M146a. The results showed significantly elevated levels of the M2 microglia markers Arg1, IL-10, CD206, and TGF-β (Figure 2B), while the pro-inflammatory cytokines IL-6, TNF-α, CD16, and IL-1β, which are produced by M1 microglia, decreased in the APP/PS1-AAV-M146a group compared with the APP/PS1-AAV-Mcon group (Figure 2C). In addition, we found that the level of the classic microglial marker Iba1 was elevated in the APP/PS1-AAV-M146a group compared with the APP/PS1-AAV-Mcon group in the brain (Figure 2D-E). However, the expression level of milk fat globule factor-E8 (MFG-E8) did not show a significant change (Figure 2F). We further detected the mRNA level of MFG-E8 in the microglia and found an increasing of MFG-E8 in the APP/PS1-AAV-M146a group compared with the APP/PS1-AAV-Mcon group (Figure 2G). We also detected the level of plaque-associated CD68, which is a marker of phagocytic activity of microglia, and found that the area of CD68 and the area ratio of CD68/Aβ_42_ were increased in the APP/PS1-AAV-M146a group compared with the APP/PS1-AAV-Mcon group (Figure 2H-J). These data revealed that miR-146a regulated microglial phenotype transition and reduced neuroinflammation in an AD mouse model.

### Microglia-specific miR-146a overexpression reduced Aβ levels and amyloid plaques in an AD mouse model

**Figure 3:**
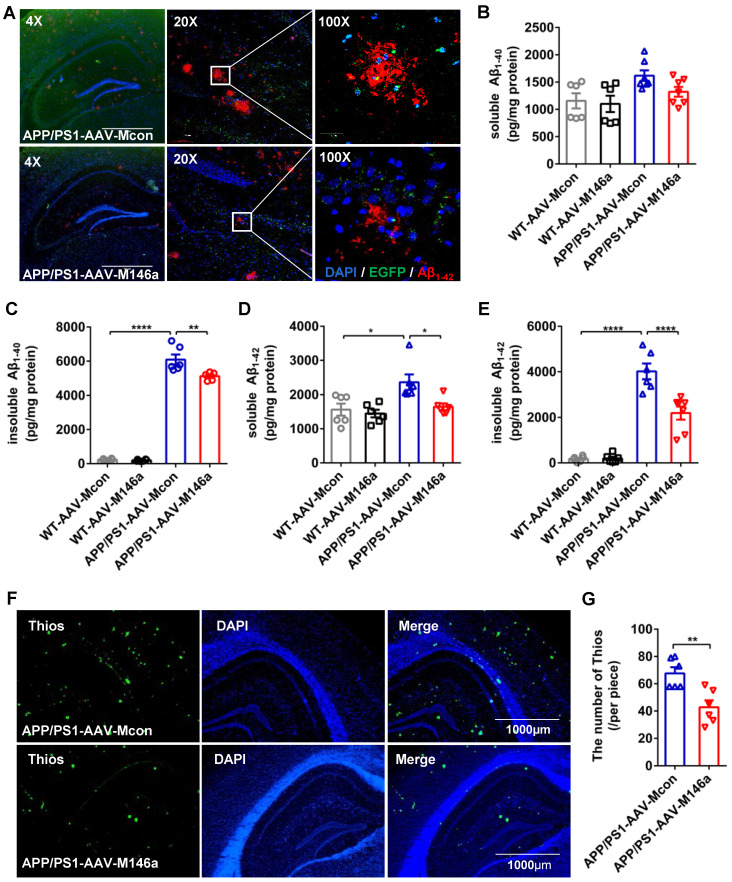
** Microglia-specific miR-146a overexpression reduced Aβ levels and amyloid plaques in the APP/PS1 mouse model.** A: Representative images of total Aβ_42_ deposition levels in the hippocampus of 11-month-old mice with Aβ_42_ (red) staining. Scale bar: 1000 μm. B-E: The expression levels of soluble or insoluble Aβ_40_ and soluble or insoluble Aβ_42_ in each group were detected by ELISA (n=6). Statistical analysis was performed using one-way ANOVA followed by post hoc Tukey's test for multiple comparisons. F: Representative images of amyloid plaque staining with Thios (green) and DAPI-immunolabelled nuclei in APP/PS1-AAV-M146a mice and APP/PS1-AAV-Mcon mice. Objective magnification: 4X, scale bar: 1000 μm. G: The number of Thios staining was quantified in APP/PS1-AAV-M146a mice and APP/PS1-AAV-Mcon mice. Data are shown as the means ± S.E.M. and were analysed by two-tailed unpaired t test with the Mann-Whitney test. *P<0.05, **P<0.01, ***P<0.005, and ****P<0.001.

Aβ is one of the classic pathological features of AD, and the polarization of microglia in AD is closely related to the phagocytosis of Aβ. Staining of Aβ with an Aβ_42_ antibody in hippocampus sections revealed a significant reduction in Aβ load in the APP/PS1-AAV-M146a group compared with the APP/PS1-AAV-Mcon group (Figure 3A). We next measured the levels of soluble and insoluble Aβ_40_ and Aβ_42_ in whole brain lysates using ELISA, and the results showed that the levels of insoluble Aβ_40_ and soluble and insoluble Aβ_42_ were significantly decreased in the APP/PS1-AAV-M146a group compared to the APP/PS1-AAV-Mcon group (Figure 3C-E), but the level of soluble Aβ_40_ was not changed (Figure 3B). Furthermore, we used Thios staining to detect whether AAV-M146a treatment influenced plaque formation in APP/PS1 mice. The results showed that compared with AAV-Mcon, AAV-M146a reduced plaque formation (Figure 3F-G). Taken together, our results demonstrated that microglia-specific miR-146a overexpression ameliorated the Aβ level and amyloid plaques in this AD mouse model.

### Microglia-specific miR-146a overexpression reduced plaque-associated neuritic pathology and neuronal loss in an AD mouse model

**Figure 4:**
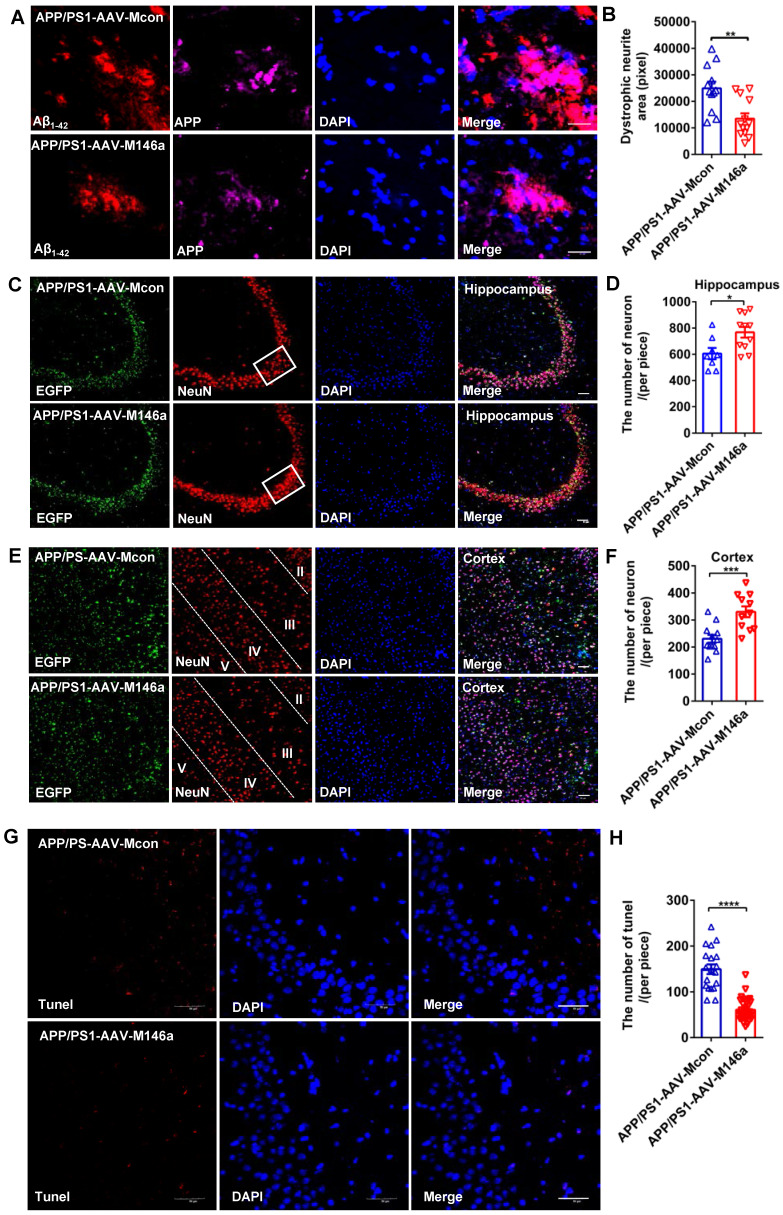
** Microglia-specific miR-146a overexpression reduced plaque-associated neuritic pathology and neuronal loss in an AD mouse model.** A: Representative images of APP-immunolabelled dystrophic neurites (purple) surrounding plaques co-labelled for Aβ_42_ (red) in the hippocampus of APP/PS1-AAV-M146a mice and APP/PS1-AAV-Mcon mice. Scale bar: 20 μm. B: The total area of dystrophic neurites was quantified in the hippocampus of APP/PS1-AAV-M146a mice and APP/PS1-AAV-Mcon mice. C: Representative images of NeuN-immunolabelled neurons (red) surrounding microglia and DAPI-immunolabelled nuclei in the hippocampus of APP/PS1-AAV-M146a mice and APP/PS1-AAV-Mcon mice. Scale bar: 50 μm. D: Quantification of neurons in the hippocampus of APP/PS1-AAV-M146a mice and APP/PS1-AAV-Mcon mice using ImageJ software. E: Representative images of NeuN-immunolabelled neurons (red) surrounding microglia and DAPI-immunolabelled nuclei in the cortex of APP/PS1-AAV-M146a mice and APP/PS1-AAV-Mcon mice. Scale bar: 20 μm. F: Quantification of neurons in the cortex of APP/PS1-AAV-M146a mice versus APP/PS1-AAV-Mcon mice using ImageJ. G: Representative images of neural apoptosis with TUNEL staining and DAPI-immunolabelled nuclei in the hippocampus of APP/PS1-AAV-M146a mice and APP/PS1-AAV-Mcon mice. Objective magnification: 40X, scale bar: 50 μm. H: Quantification of apoptotic cells in the hippocampus of APP/PS1-AAV-M146a mice and APP/PS1-AAV-Mcon mice using ImageJ software. Data are shown as the means ± S.E.M. and were analysed by two-tailed unpaired t test with the Mann-Whitney test for B, D, F and H. *P<0.05, **P<0.01, ***P<0.005, and ****P<0.001.

Previous studies have reported that neurite dystrophy is closely associated with Aβ deposits in AD patients and mouse models, and whether this phenotype is alleviated by miR-146a overexpression in an AD mouse model has not yet been studied. To directly assess plaque-associated neurite dystrophy, we performed immunostaining with an antibody against the N-terminus of APP, which accumulates in the damaged neurites. Our analyses showed that dystrophic neurites were reduced in the APP/PS1-AAV-M146a group compared to the APP/PS1-AAV-Mcon group (Figure 4A-B), which provided further evidence that microglia-specific miR-146a overexpression conferred a neuroprotective effect *in vivo*. Moreover, NeuN immunostaining was performed to evaluate potential neuronal protection. As shown in Figure 4 C-E, we observed an increased number of neurons in the hippocampus (Figure 4C-D) and cortex (Figure 4E-F) in the APP/PS1-AAV-M146a group compared with the APP/PS1-AAV-Mcon group. Furthermore, TUNEL staining was used to detect whether treatment with AAV-M146a suppressed neural apoptosis in APP/PS1 mice. The results showed that compared with AAV-Mcon, AAV-M146a reduced neural apoptosis (Figure 4G-H), which indicated that microglia-specific miR-146a overexpression can decrease neural apoptosis in the AD mouse model. In summary, the above results suggested that microglia-specific miR-146a overexpression controlled neuronal survival in an AD mouse model.

### Increased miR-146a levels trigger a switch in the microglial phenotype in an Aβ_42_- or LPS-induced cell model

**Figure 5:**
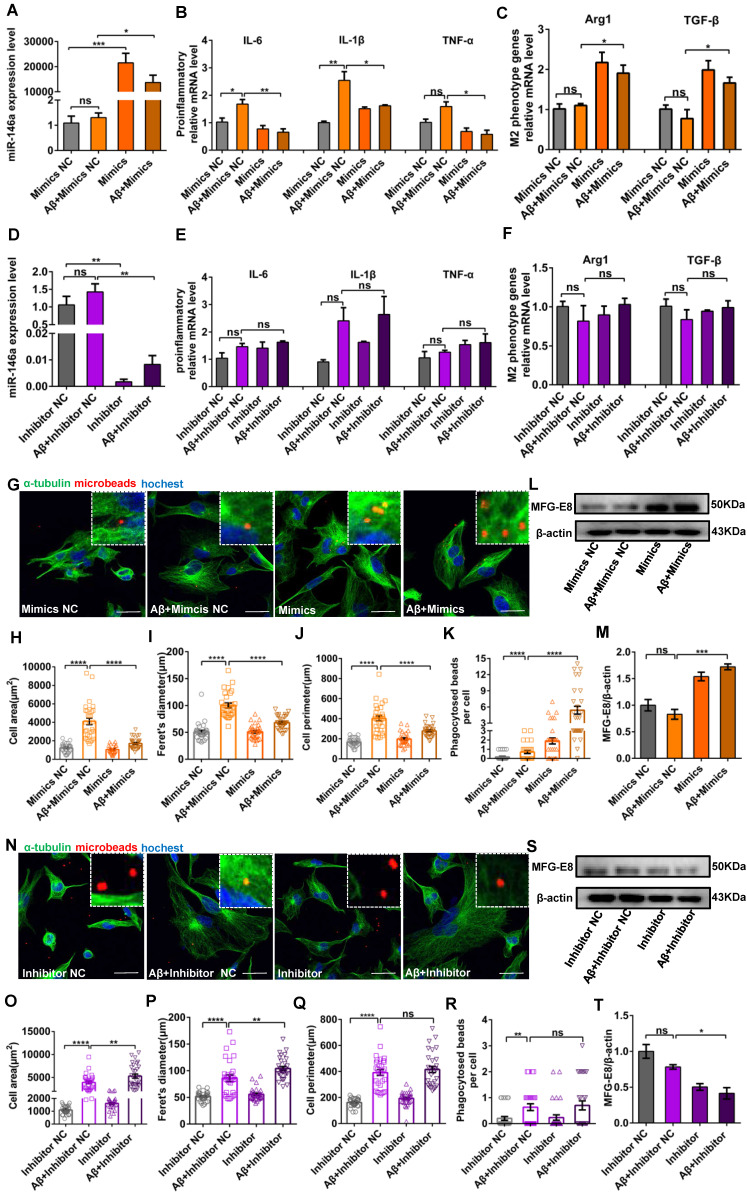
** Increased miR-146a triggered microglial phenotype switching and enhanced phagocytosis in Aβ_42_-treated cells.** HMC3 cells were transfected with 10 μM miR-146a mimics/100 μM inhibitor and given 5 μM Aβ_42_ for 12 h. A: The level of miR-146a was increased by overexpressing exogenous miR-146a-5p. Statistical analysis was performed using one-way ANOVA followed by post hoc Tukey's test for multiple comparisons. B: The mRNA levels of pro-inflammatory factors (IL-6, IL-1β and TNF-α) secreted by M1 phenotype microglia were detected by qRT-PCR. Statistical analysis was performed using two-way ANOVA followed by post hoc Tukey's test for multiple comparisons. C: Quantification of the mRNA levels of M2 phenotype microglia markers (Arg1 and TGF-β) was performed by qRT-PCR. Statistical analysis was performed using two-way ANOVA followed by post hoc Tukey's test for multiple comparisons. D: The level of miR-146a decreased upon the expression of exogenous miR-146a-5p inhibitor. Statistical analysis was performed using one-way ANOVA followed by post hoc Tukey's test for multiple comparisons. E: The mRNA levels of IL-6, IL-1β and TNF-α were detected by qRT-PCR. F: Quantification of the mRNA levels of Arg1 and TGF-β was performed by qRT-PCR. Two-way ANOVA with Tukey's multiple comparisons test was used for E and F. ns: no significance. G and N: HMC3 cells were treated as described above and stained with α-tubulin (green) to visualize the cytoskeleton. After a 3 h incubation, phagocytosed microspheres appeared red, and DAPI-stained nuclei appeared blue. Objective magnification: 20X, zoom: X4, scale bar: 30 μm. H-K and O-R: The cell area, 'Feret diameter, circumference and phagocytosis of fluorescent beads were measured by ImageJ in the miR-146a mimic group (H-K) and inhibitor group (O-R). Statistical analysis was performed using one-way ANOVA followed by post hoc Tukey's test for multiple comparisons. L and S: The level of MFG-E8 measured by Western blot analysis in the miR-146a mimic group (L) and inhibitor group (S). M and T: Quantification of the MFG-E8 levels in the miR-146a mimic group (M) and inhibitor group (T) was performed with ImageJ. Statistical analysis was performed using one-way ANOVA followed by post hoc Tukey's test for multiple comparisons. The data shown are the means ± S.E.M. from three independent experiments. *P<0.05, ** P<0.01, *** P<0.005, and **** P< 0.001.

After confirming that miR-146a overexpression transformed microglia from the M1 to M2 state *in vivo*, we further confirmed these results and tried to determine the association between miR-146a and the inflammatory response of microglia *in vitro*. First, we investigated the markers of microglial polarization after miR-146a overexpression. We found that the mRNA expression of the pro-inflammatory cytokines TNF-α, IL-1β, and IL-6, which are produced by M1 microglia, was significantly downregulated in the miR-146a mimic groups compared to the mimic NC groups under Aβ_42_ induction (Figure 5A-B) or under LPS induction (Figure S2A-B). In contrast, the M2 microglial marker Arginase 1 (Arg1) and the anti-inflammatory cytokine TGF-β, which can antagonize the M1 pro-inflammatory responses that ultimately result in immunosuppression and neuroprotection, were significantly increased in the miR-146a groups compared to the mimic NC groups under Aβ_42_ induction (Figure 5C) or under LPS induction (Figure S2C). Conversely, there was no significant decline in TNF-α, IL-1β, or IL-6 in the miR-146a inhibitor groups compared to the inhibitor NC groups under Aβ_42_ induction (Figure 5D-E) or under LPS induction (Figure S2D-E). Similarly, no obvious differences were detected in the mRNA levels of Arg1 or TGF-β between miR-146 inhibition and non-inhibition under neuroinflammation caused by Aβ_42_ (Figure 5F) or under LPS stimulation (Figure S2F). The above results suggested that miR-146a can trigger microglia to decrease pro-inflammatory phenotypes and enhance phagocytic phenotypes in response to Aβ_42_ and LPS induction.

### Increased miR-146a levels enhance microglial phagocytosis in an Aβ_42_- or LPS-induced cell model

Then, we determined whether miR-146a overexpression can raise microglial phagocytosis. Microglia treated with LPS or Aβ_42_ showed an amoeboid phenotype, and their area, Feret's diameter and circumference were significantly higher than those of control microglia (Figure 5G-J and Figure S2G-J). However, when the level of miR-146a in microglia was increased prior to the administration of Aβ_42_, the microglia were circular or fusiform, and the area, Feret's diameter and circumference decreased compared to the mimic NC group (Figure 5G-J). Similar results were also obtained when microglia were treated with LPS (Figure S2G-J). In contrast, when miR-146a expression was inhibited before Aβ_42_-induced neuroinflammation, the area, Feret's diameter and circumference of the microglia did not significantly decrease compared to those of the mimic NC group (Figure 5N-Q). Similar results were obtained when the stimulus was changed from Aβ_42_ to LPS (Figure S2N-Q). Furthermore, polystyrene microbeads were used to assess the general phagocytic activity of microglia and to ensure that phagocytosis was measured rather than pinocytosis, and the results showed a significant enhancement of phagocytic activity in the miR-146a-overexpressing group compared to the mimic NC-expressing group (Figure 5K), but the miR-146a inhibitor-expressing microglia showed nonsignificant phagocytic activity under Aβ_42_-induced phagocytic activity (Figure 5R). Similar results were also obtained when the stimulus was changed from Aβ_42_ to LPS (Figure S2K and R). In addition, milk fat globule factor-E8 (MFG-E8), a key factor mediating macrophage phagocytosis of apoptotic cells, was also assessed, and the results showed no significant change in the level of MFG-E8 after Aβ_42_ stimulation, but the miR-146a mimics increased it (Figure 5L-M). In contrast, when miR-146a expression was inhibited before Aβ_42_ stimulation, it also did not increase the protein level of MFG-E8 (Figure 5S-T), and similar results were also obtained under LPS stimulation (Figure S2L-M and S-T). In conclusion, these results showed that the miR-146a mimic restored the phagocytosis of microglia under stimulation.

### Increased miR-146a levels in microglia prevent Aβ_42_- or LPS-induced apoptosis of neurons

**Figure 6:**
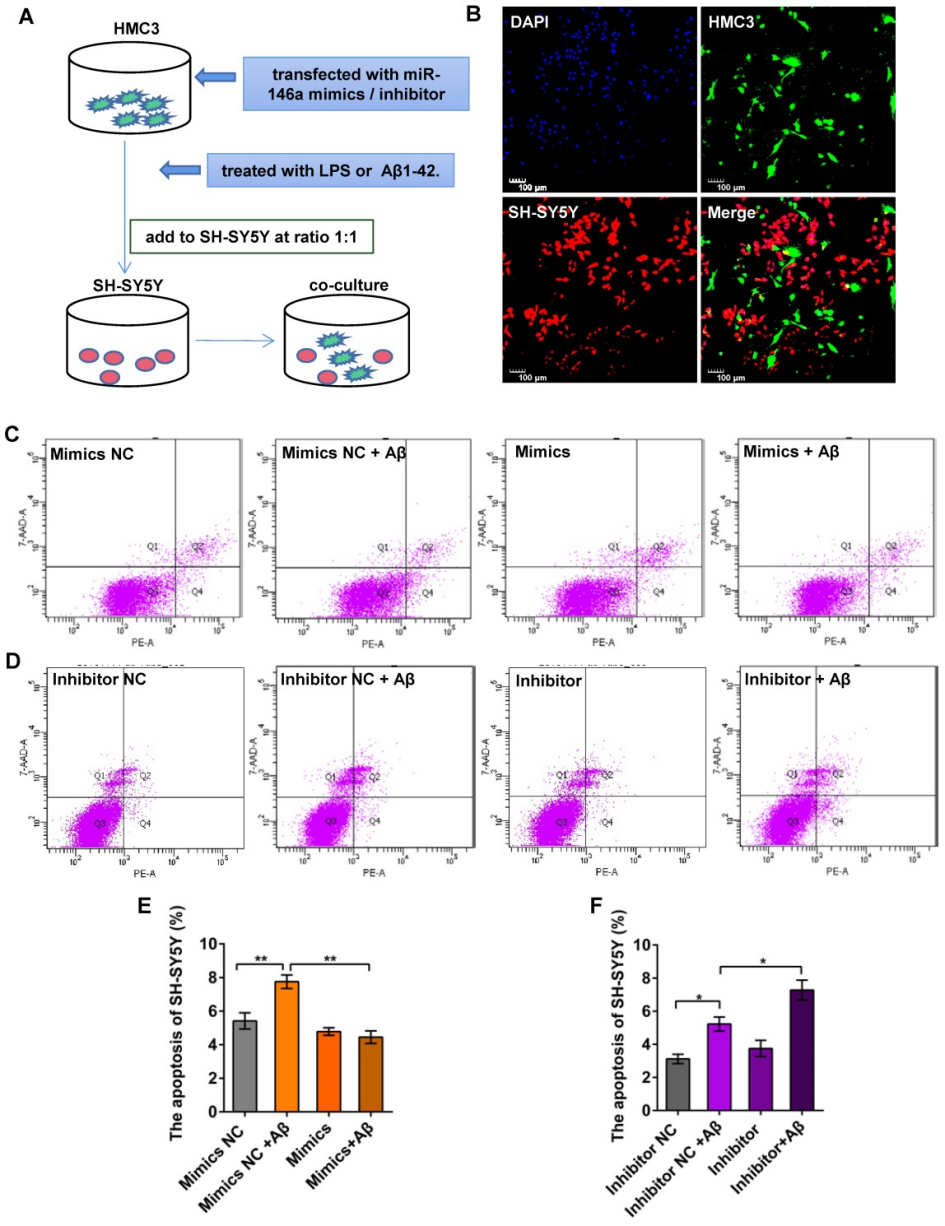
** Increased miR-146a in microglia prevented neuronal apoptosis in Aβ_42_-treated cells.** A: HMC3 cells were transfected with 10 μM miR-146a mimics/100 μM inhibitor and treated with 5 μM Aβ_42_ for 12 h. SH-SY5Y cells were then added to the HMC3 cells at a 1:1 ratio, and apoptosis was detected by flow cytometry after 24 h. B: HMC3 cells were infected with GFP-adenovirus, and SH-SY5Y cells were stained with CellTrace Far Red. C-D: The apoptosis of SH-SY5Y cells was analysed by flow cytometry in the miR-146a mimics group (C) and inhibitor group (D). E-F: Aβ_42_ treatment of HMC3 cells increased the apoptosis of SH-SY5Y cells, an effect that was reduced by miR-146a mimics (E) but increased in the presence of miR-146a inhibitor (F). Statistical analysis was performed using one-way ANOVA followed by post hoc Tukey's test for multiple comparisons. Data are shown as the means ± S.E.M from four independent experiments. *P<0.05, ** P<0.01, *** P<0.005, and **** P<0.001.

To determine whether miR-146a regulated the phenotypes of microglia, thereby reducing neuronal damage, we first treated HMC3 cells with miR-146a mimics or inhibitor *in vitro* and then added Aβ_42_ or LPS to activate microglia. In turn, we co-cultured HMC3 and SH-SY5Y cells directly at a 1:1 ratio to evaluate neuronal apoptosis (Figure 6A-B). We used an Annexin V-PE/7-AAD Apoptosis Kit and flow sorting, and our results showed that the apoptosis rate of SH-SY5Y cells increased, when the HMC-3 cells were treated with Aβ_42_ (Figure 6C-D) or LPS (Figure S3A-B). However, the apoptosis rate of the SH-SY5Y cells declined in the miR-146a mimic group compared with the mimic NC group upon stimulation with Aβ_42_ (Figure 6C and E) or LPS (Figure S3A and C). However, when the HMC3 cells were treated with a miR-146a inhibitor and stimulated with Aβ_42_, the apoptosis rate of the SH-SY5Y cells increased instead of decreasing (Figure 6D and F). Similarly, consistent results were obtained when the stimulus was changed from Aβ_42_ to LPS (Figure S3 B and D). These results showed that miR-146a reduced the damage to neurons caused by Aβ_42_- or LPS-induced microglia and acted as a protector of neurons.

### Neuroinflammation-associated gene signatures in the brain with microglial miR-146a overexpression were identified

**Figure 7:**
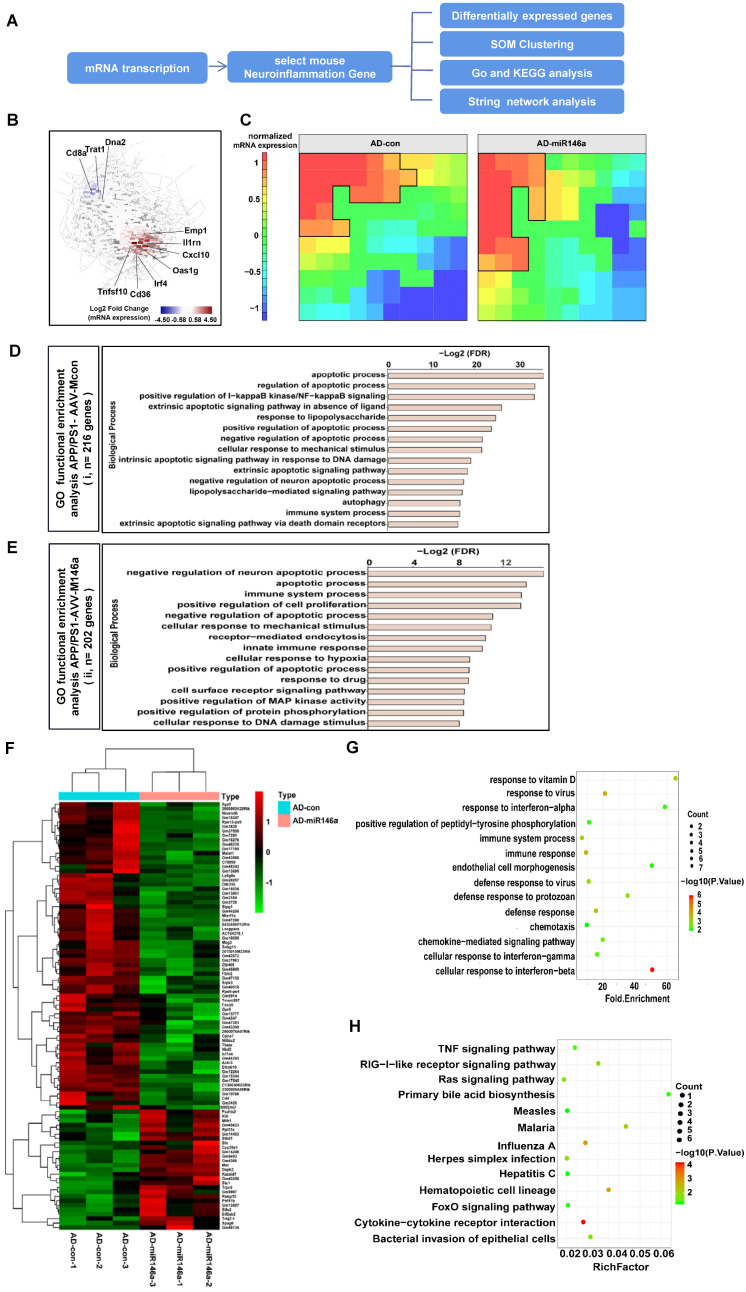
** Neuroinflammatory gene signatures were identified by transcriptional analysis in the brains of the microglial miR-146a-overexpressing AD mouse model.** A: Workflow for neuroinflammatory gene analysis. B: Gene network analysis of regulated genes in the APP/PS1-AAV-Mcon group and APP/PS1-AAV-M146a group identified by transcriptional analysis. C: SOM clustering of the APP/PS1-AAV-Mcon group and APP/PS1-AAV-M146a group with the definition of gene sequences. D-E: Gene signatures in APP/PS1-AAV-Mcon mice defined by cluster (i) and in APP/PS1-AAV-M146a mice defined by cluster (ii). Fisher's exact test followed by a correction for multiple testing. F: Heatmap of differential genes. The differentially expressed genes between the APP/PS1-AAV-Mcon group and the APP/PS1-AAV-M146a group were obtained by transcriptional analysis. G: The differentially expressed genes were analysed by GO gene ontology enrichment to obtain the biological processes. H: The pathway enrichment analysis of differential genes was performed using the KEGG database (https://www.kegg.jp/).

To explore the effects of microglial miR-146a overexpression on metabolic pathways, especially on the neuroinflammation-related signalling pathways of AD, we analysed the transcriptional data of the cerebral samples of APP/PS1-AAV-M146a and APP/PS1-AAV-Mcon mouse group (Figure 7A). We visualized the dynamics of gene expression patterns in a co-regulatory network. As shown in Figure 7B, several inflammatory-related genes, including Oas1g, CD36 and Cxcl10, were among the top upregulated genes, indicating a central role of miR-146a in neuroinflammation. Self-organizing map (SOM) clustering also identified genes that participate in neuroinflammation (Figure 7C). The genes were grouped according to similar expression levels in the APP/PS1-AAV-M146a and APP/PS1-AAV-Mcon mouse groups. Gene set enrichment analysis (GSEA) of highly expressed genes in the APP/PS1-AAV-M146a mouse group showed enrichment in biological processes such as “negative regulation of neuron apoptotic process” and “immune system process” (Figure 7E), while those in the AD control mouse group showed “apoptotic process” (Figure 7D). Then, we visualized the central highly interacting genes that might regulate or collaborate with other genes during disease development as a network. The network for the genes upregulated in the APP/PS1-AAV-M146 mice showed two clusters of highly interactive genes: one cluster associated with Fyn signalling cascades and another cluster consisting of Trp53, indicating that the function of microglia in APP/PS1-AAV-M146 mouse pathology involves apoptosis (Figure S4B). In contrast, the network for the genes with higher expression in the APP/PS1-AAV-Mcon mice was less dense, and the central genes were TNF, MyD88, and TRADD, which indicates an inflammatory phenotype of Aβ pathology (Figure S4A). Next, we analysed the gene functions of the genes that were altered between the APP/PS1-AAV-Mcon group and APP/PS1-AAV-M146 group to comprehensively elucidate the possible gene-regulated pathways involved in the protective effect of miR-146a in microglia that inhibits the development of AD. The results showed that 69 downregulated and 53 upregulated genes were identified in the APP/PS1-AAV-M146 group compared to the APP/PS1-AAV-Mcon group (Figure 7F). We observed that in addition to the defence response against viruses, the biological process functions of the differentially expressed genes focused on “immune response”, “immune system process”, “defense response”, and “cell response to interferon” (Figure 7G), which suggested that miR-146a is of great importance to the immune response in the development of AD. Moreover, we analysed the potential pathways based on the differentially expressed genes and found that 13 pathways, including cytokine-cytokine receptor interaction and the Ras and TNF signalling pathways, were significantly involved in microglia-specific miR-146a overexpression (Figure 7H), suggesting that miR-146a had a multi-pathway therapeutic effect on AD. Based on the transcriptome analysis, we confirmed that specific miR-146a overexpression indeed improved the protective functions of microglia under AD pathological conditions and changed the immune response pathway in the brain of an AD mouse model.

### Identification and validation of miR-146a target genes

Next, to identify target genes playing important roles in AD, we further screened the downregulated genes in the miR-146a upregulated groups to identify potential miR-146a targets (Figure S5A). Ultimately, four genes (FoxO6, Nkd2, Cd4 and Stpg1) were identified as having potential binding sites in the 3' UTR of miR-146a (Figure S5B). Dual-luciferase reporter assays were performed to confirm whether the screened genes were miR-146a targets. The results showed that the fluorescence intensity was reduced with the “565-571” binding region of Stpg1 and the “1882-1888” binding region of Nkd2 under miR-146a mimic administration in 293T cells, suggesting that miR-146a directly binds to the 3' UTRs of the Stpg1 gene and Nkd2 gene, while the 3' UTRs of FoxO6 and Cd4 were not significantly altered (Figure S5C). Then, we measured the mRNA and protein levels of Nkd2 and Stpg1 *in vivo* and found that compared with those in the APP/PS1-AAV-Mcon group, the mRNA levels of Nkd2 and Stpg1 were downregulated in the APP/PS1-AAV-M146a group, but unfortunately, their protein levels did not decrease (Figure S5D-H). Moreover, to determine whether the mRNA level of target genes could be changed by miR-146a regulation *in vitro*, this correlation was further validated in 293T cells (Figure S5I-J) and HMC3 cells (Figure S5K-L). The results showed that the mRNA level of Nkd2 was negatively regulated by miR-146a, whereas Stpg1 did not show a change *in vitro* (Figure S5I-L).

We further examined whether knockdown of Nkd2 affected the microglial phenotype under Aβ stimulation *in vitro* and found that the knockdown of Nkd2 reduced the expression of IL-6 and IL-1β and elevated the expression of Arg1 and IL-10 under Aβ stimulation (Figure S5M-Q). Then, we studied whether knockdown of Nkd2 could reverse the effects of miR-146a inhibition on the microglial phenotype. The results showed that knockdown of Nkd2 reversed the effects of miR-146a inhibition on IL-1β and IL-10 expression but not on IL-6 and Arg1 expression (Figure S5R-U). Therefore, we concluded that the knockdown of Nkd2 showed a significant effect on the change in microglial phenotype under Aβ stimulation but did not reverse or block the effect of miR-146a inhibition on the phenotype of microglia.

## Discussion

In the present study, we reported that microglia-specific overexpression of miR-146a effectively prevented cognitive impairment and the main pathological process in an AD mouse model. Furthermore, we confirmed *in vivo* and *in vitro* that this beneficial effect was achieved by influencing the phenotypes of microglia, highlighting the mechanism of miR-146a in rescuing the phenotype and pathology of AD and its potential as a target for microglia. For a long time, research on the mechanism and therapeutic targets of AD has focused on the two most important hallmarks, Aβ and Tau, but results from clinical studies in AD are not encouraging 6, 20. It is worth noting that an excessive immune response and chronic neuroinflammation have been considered downstream pathological processes of AD. Increasing evidence strongly supports that microglia, as the main immune cells in the neuroimmune inflammatory response, are the key target to inhibit Aβ and/or Tau-induced neuronal injury in AD 1, 21. Solid evidence has shown that Aβ activates microglia and induces neuroinflammation in AD progression 22, 23. Microglia-Aβ interactions lead to early synapse loss, production of neurotoxic reactive oxygen and nitrogen species (ROS and RNS), NLRP3 inflammasome activation, and production of pro-inflammatory cytokines and TNF27 24-27. Notably, the misfolded and aggregated Aβ protein can be phagocytosed and cleared by activated microglia 6, and microglial depletion leads to an increased plaque burden 28, suggesting that microglia have a critical role in AD and are a potential therapeutic target to reduce the Aβ level in AD.

Microglia are thought to act as a “double-edged” sword to provide beneficial or harmful effects in the central nervous system depending on phenotypic polarization. Activated microglia can be divided into M1 and M2 categories through different gene expression patterns, which also represent different microglial functions 29. Excessive activation of M1 microglia exacerbates AD pathological damage, as shown by a suite of phenotypic markers specific to pro-inflammatory conditions 30-32. Correspondingly, M2 microglia exert neuroprotective effects by releasing anti-inflammatory cytokines, reducing the spread of Aβ products, markedly alleviating neuroinflammatory responses, and ultimately ameliorating pathological impairment in AD 33-35. However, microglia are considered to have multiple reactive phenotypes related to the type and stage of AD 36, 37. The changes in the phenotypes of microglia are complicated and may differ with the stage and severity of AD. Therefore, in the present study, we mainly evaluated the effect of miR-146 overexpression on the pro-inflammatory phenotype and phagocytic phenotype of microglia, which is the classic phenotype associated with AD pathology.

Our group previously showed that the administration of miR-146a reduced the main pathological processes of AD, including glial activation and neuroinflammation, in APP/PS1 transgenic mice, highlighting its potential as a therapeutic target for AD 18. In the present study, we focused on the effect of miR-146a on microglial function under AD conditions. Our results showed that upregulation of the level of microglia-specific miR-146a reduced cognitive impairment in an AD mouse model. Additionally, it attenuated neuroinflammation and Aβ levels and prevented neuronal loss. These results were consistent with those of our previous report, in which nonspecific brain administration of a miR-146a agomir based on the naso-brain pathway system rescued AD pathology 18, supporting the conclusion that miR-146a overexpression in microglia is a crucial strategy to treat the affected cell types in AD.

We further confirmed that miR-146a overexpression switched microglial polarization. We found that miR-146a upregulation in microglia changed the state of microglia in AD pathology to the “good” phagocytic phenotype. To further confirm these results, we evaluated the function of microglia after miR-146a overexpression *in vitro*. Here, two cell models were used to model microglial activation *in vitro*: an Aβ-induced model and an LPS-induced model. Aβ is known to accelerate AD through neuronal damage induced by the expression of inflammatory mediators 38, 39. Accordingly, LPS-provoked neuroinflammation can cause synaptic loss and induce cognitive impairment in AD 40, 41 and is also associated with the enhancement of Aβ production in an AD mouse model 40, 42, 43. Thus, both Aβ and LPS are causative factors in the pathogenesis of AD by inducing microglial activation 40, 44, 45. Our results showed that microglial miR-146a overexpression both decreased the pro-inflammatory phenotype and enhanced the phagocytic phenotype following LPS or Aβ induction, which was in accordance with the *in vivo* results. However, microglial inhibition of miR-146a did not show the opposite effect, as expected, and it did not significantly transform microglial polarization. These unexpected negative results may be related to the low endogenous expression of miR-146a under the stimulation conditions; reduction in the miR-146a level may not be enough to produce significant functional changes in microglia. On the other hand, under normal physiological conditions, the microglia existed in a resting state and did not show phagocytosis or a pro-inflammatory state caused by miR-146a overexpression in the absence of external stimulation. This may also suggest that miR-146a has a role in the functional transformation of microglia but may not have an effect on the activation of microglia. This mechanism will be explored in the future. In summary, we conclude that the main effect of miR-146a on microglia is anti-inflammatory polarization, supporting the view that a high level of miR-146a serves as a potent inhibitor of LPS- and Aβ-induced neuroinflammation and is a microglial phenotype regulator *in vivo* and *in vitro*, which indicates that miR-146a may be a suitable therapeutic candidate in AD.

It has been reported that microglial dysfunction damages neurons in AD. This damage can be divided into direct and indirect pathways. In the direct pathway, when microglia interact with the ligand Aβ, they trigger a variety of pathways to release harmful cytokines, such as iNOS and ROS, leading to neuronal apoptosis and dysfunction. In the indirect pathway, dysfunctional microglia may release pro-inflammatory factors, such as TNF-α, or reduce the production of the neuroprotective protein brain-derived neurotrophic factor (BDNF) or insulin-like growth factor (IGF), thereby increasing neuronal apoptosis 8. After we identified the beneficial regulatory effect of miR-146a on the function of microglia in AD, we further evaluated the damage to neurons after miR-146a overexpression in microglia of the AD model mice, and the results showed protection of neurons in layer IV of the cortex and the CA3 zone of the hippocampus *in vivo*, which implied that miR-146a hindered destructive pro-inflammatory responses and promoted protective phagocytic responses in microglia, thereby protecting neighbouring neurons directly or indirectly. Then, we also demonstrated *in vitro* that microglial administration affected neuronal damage under LPS and Aβ induction.

We also explored the pathological pathways affected by miR-146a overexpression in microglia of AD model mice. Transcriptional analysis showed that the biological process functions related to the differentially expressed genes focused on “immune response”, “immune system process”, and “defense response”, which suggested that miR-146a is of great importance to the immune response in the development of AD. Moreover, we analysed the potential pathways based on the differentially expressed genes and found that 13 pathways, including cytokine-cytokine receptor interaction and the Ras and TNF signalling pathways, were significantly involved in increased miR-146a in microglia, suggesting that the therapeutic effects of miR-146a on AD may be achieved through multiple pathways. Furthermore, we wanted to identify the genes that were directly regulated by miR-146a and led to changes in the microglial phenotype in the AD pathological state. Through bioinformatics analysis, we identified four candidates, confirmed that Stpg1 and Nkd2 directly interacted with miR-146a, and found that compared with those in the APP/PS1-AAV-Mcon group, the mRNA levels of Nkd2 and Stpg1 were downregulated in the APP/PS1-AAV-M146a group. We further proved that only the mRNA level of Nkd2 was negatively regulated by miR-146a *in vitro*. Moreover, knockdown of Nkd2 showed a significant effect on the change in microglial phenotype under Aβ stimulation *in vitro*, but it did not reverse or block the effect of miR-146a inhibition on the phenotype of microglia. Based on our previous transcriptome analysis, we confirmed that specific miR-146a overexpression indeed made microglial function more beneficial under AD pathological conditions and changed the immune response pathway in the brain of an AD mouse model. A specific molecular mechanism will be explored in the future.

Several limitations of this study should be addressed. First, we only identified that microglia-specific overexpression with miR-146a is definitely beneficial for AD treatment. MiR-146a is not only expressed in microglia but may also have potential functions in astrocytes 46-48, neurons 49-51, oligodendrocytes 52, and endothelial cells 53. However, instead of focusing on all neurocyte subtypes, our results demonstrated the effects of miR-146a in microglia on AD progression. Second, we identified that miR-146a can change microglial polarization and proposed several potentially important pathways through transcriptome analysis. However, the specific pathways and targets involved in AD progression were only preliminarily validated, and the specific mechanisms and downstream targets as well as the potential effects of miR-146a on other neuronal cells will be investigated in future studies.

## Conclusion

In summary, we reported that microglial-specific miR-146a overexpression can be a new valuable therapy for AD. A high level of microglia-specific miR-146a expression reduced cognitive deficits in learning and memory, attenuated neuroinflammation, reduced Aβ levels, and prevented neuronal loss in APP/PS1 Tg mice. Notably, miR-146a has been shown to increase microglial transformation, decrease the pro-inflammatory phenotype, increase the phagocytic phenotype, and protect neurons under AD conditions. Our results provide evidence that miR-146a is a promising target for AD and other microglia-related diseases. We will further explore the in-depth mechanism of miR-146a in microglial polarization as well as perform pharmacological research for clinical practice.

## Supplementary Material

Supplementary figures and tables.

## Abbreviations

AD: Alzheimer's Disease
NLRP3: NLR Family Pyrin Domain Containing 3
ASC: Activating Signal Cointegrator
IL-6: Interleukin 6
TNF: Tumor Necrosis Factor
IL-1β: Interleukin 1 Beta
Arg1: Arginase 1
TGF-β: Transforming Growth Factor Beta
MFG-E8: Milk Fat Globule EGF And Factor V/VIII Domain Containing
APP: Amyloid Beta Precursor Protein
PS1: Presenilin 1
LPS: Lipopolysaccharide
Nkd2: NKD Inhibitor Of WNT Signaling Pathway 2
Stpg1: Sperm Tail PG-Rich Repeat Containing 1
FoxO6: Forkhead Box O6
Cd4: CD4 Antigen
NF-κB: Nuclear Factor Kappa B Subunit 1
CFH: Complement Factor H
IL-1: Interleukin -1
IRAK1: Receptor-associated kinase 1
TRAF6: Receptor-associated Factor 6
UTR: Untranslated Region
Oas1g: 2'-5'-Oligoadenylate Synthetase 1
CD36: CD36 Antigen
Cxcl10: C-X-C Motif Chemokine Ligand 10
Fyn: FYN Proto-Oncogene, Src Family Tyrosine Kinase
Trp: Tumor Protein P53
MyD88: MYD88 Innate Immune Signal Transduction Adaptorand
TRADD: TNFRSF1A Associated Via Death Domain
GFAP: Glial Fibrillary Acidic Protein
Thios): Thioflavine S
