# Supplementary material for: MicroRNA-146a switches microglial phenotypes to resist the pathological processes and cognitive degradation of Alzheimer's disease
Source: Theranostics. 2021 Feb 19;11(9):4103–21. doi: 10.7150/thno.53418 (PMC7977456; doi:10.7150/thno.53418)
Supplement: Supplementary file 1 — Supplementary figures and tables. [file thnov11p4103s1.pdf]

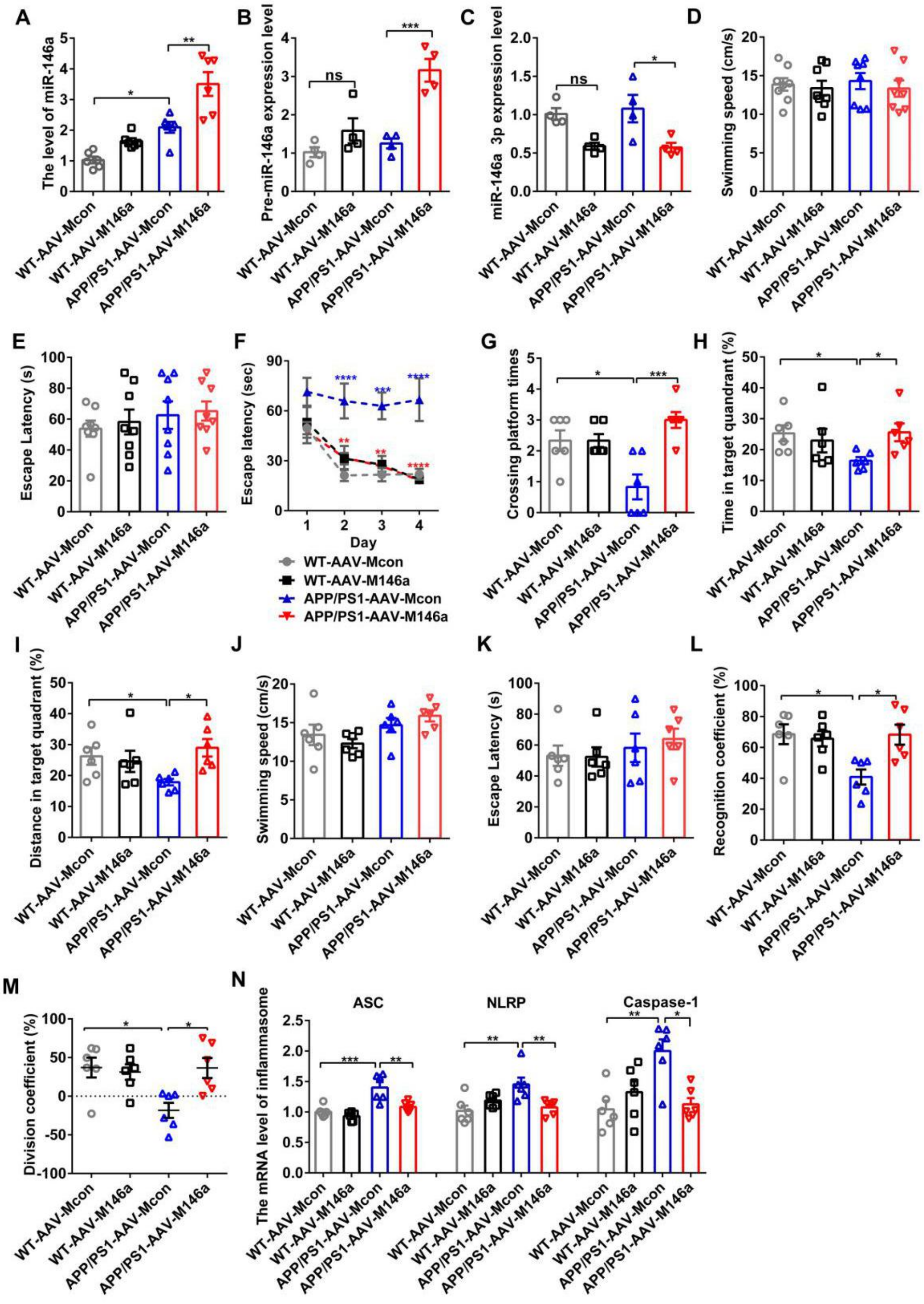

Figure S1. Microglia-specific miR-146a overexpression improved cognitive deficits in learning and memory in APP/PS1 Tg mice. A-C: Quantification of miR146a, pre-miR146a and miR146a-3p by qRT-PCR of brain (n=6 for each group). Statistical analysis was performed using one-way ANOVA followed by post hoc Tukey's tests for multiple comparisons. D-E: Quantification of swimming speed and escape latency of MWM on the first day (For each group, n=8). F: The escape latencies of each group were tested in the Morris water maze for 5 consecutive days. Statistical analysis was performed using two-way ANOVA followed by post hoc Tukey's tests for multiple comparisons (Interation: Time*Treatment, P = 0.5526, no significant effcet of two factors). G-I: Probe trials were performed at day 6, the number of times crossing in the platform site (G), the time spent in the target quadrant (H), and the more swimming distances in the target quadrant (I) are shown. Statistical analysis was performed using

one-way ANOVA followed by post hoc Tukey's tests for multiple comparisons (H-J). J-K: Quantification of swimming speed and escape latency of MWM on the first day. L-M: Quantification of recognition coefficient (L) and division coefficient (M) in the novel object recognition test of APP/PS1-AAV-M146a mice and APP/PS1-AAV-Mcon mice. one-way ANOVA with Tukey's multiple comparisons test. For each group, n = 6. N: The expression level of NLRP inflammasome (ASC, NLRP and Caspase1) was analyzed by qRT-PCR with the cerebral sample of each group (n=6). Statistical analysis was performed using Two-way ANOVA followed by post hoc Tukey's tests for multiple comparisons. Shown are the means ± S.E.M values from three independent experiments. *Represents P<0.05, **P<0.01, *** P<0.005, and ****P<0.001.

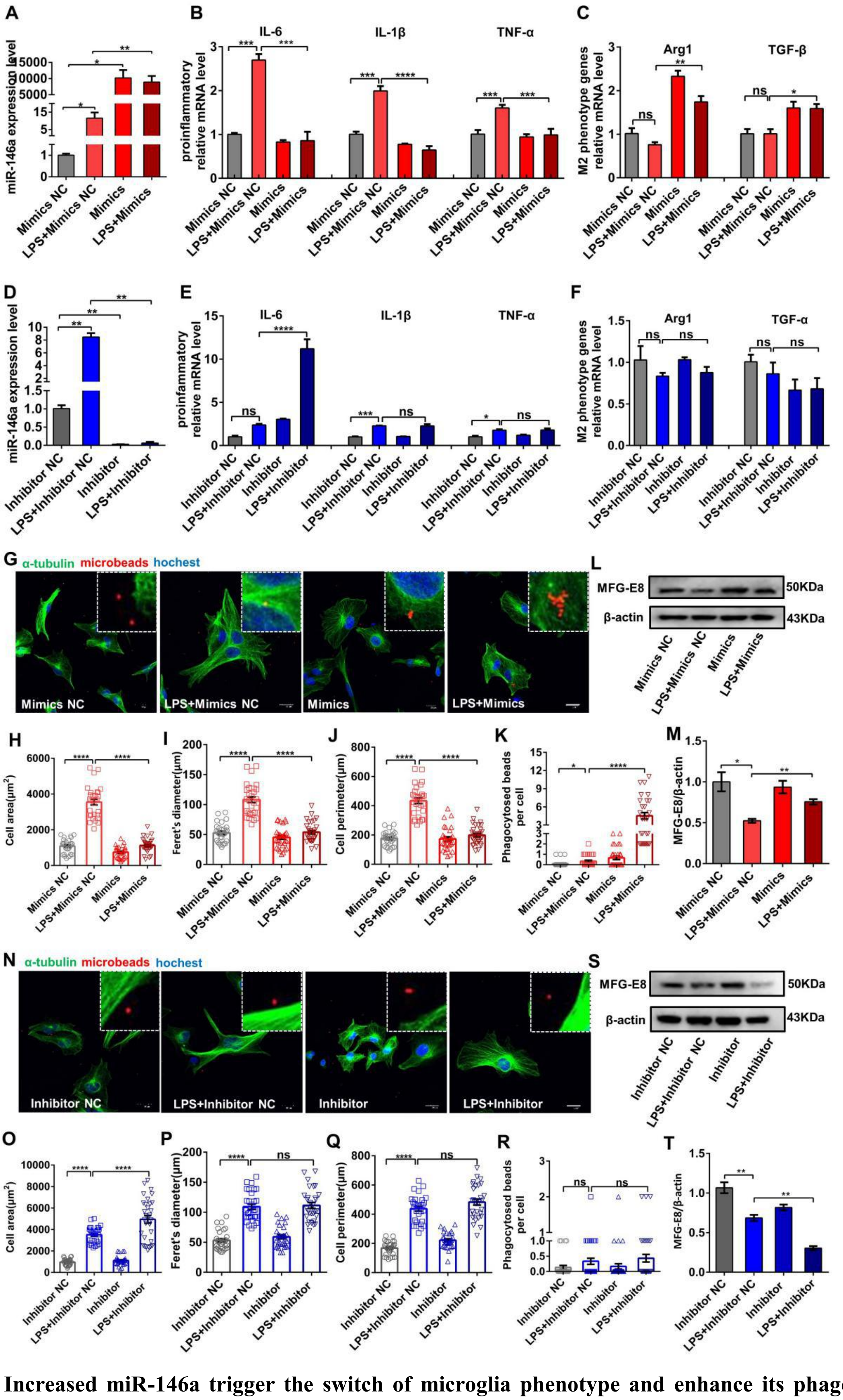

Figure S2. Increased miR-146a trigger the switch of microglia phenotype and enhance its phagocytosis in LPS induced neuroinflammation. HMC3 cells were transfected with 10 nM miR-146a Mimics /100 nM inhibitor and given 100 ng/ml LPS for 72 h. A: The level of miR-146a increased by overexpressing exogenous miR-146a.

Statistical analysis was performed using one-way ANOVA followed by post hoc Tukey's tests for multiple comparisons. B-C: mRNA levels of proinflammatory factors (IL-6, IL-1β, TNF-α) secreted by M1 phenotype microglia and M2 phenotype microglia markers (Arg1, TGF-β) were measured by qRT-PCR. Two-way ANOVA followed by post hoc Tukey's tests for multiple comparisons. D: The level of miR-146a declined by exogenous miR-146a inhibitor overexpression. Statistical analysis was performed using one-way ANOVA followed by post hoc Tukey's tests for multiple comparisons. E and F: mRNA levels of proinflammatory factors (IL-6, IL-1β, TNF-α) secreted by M1 phenotype microglia and M2 phenotype microglia markers (Arg1, TGF-β) under LPS-induced neuroinflammation with miR-146a inhibitor or not. Two-way ANOVA with post hoc Tukey's tests for multiple comparisons. ns: no significance. G and N: HMC3 were treated as above and stained with α-tubulin (green) to visualize cytoskeleton. After a 3 h incubation, phagocytosed microspheres appeared red, and DAPI-stained nuclei appeared blue. Objective magnification: 20X, Zoom: X3, Scale bar: 30 μm. H-K and O-R: The cell area, Feret's diameter, circumference and phagocytosis of fluorescent beads were measured by ImageJ. Statistical analysis was performed using one-way ANOVA followed by post hoc Tukey's tests for multiple comparisons. L-M and S-T: The level of phagocytic protein MFG-E8 was measured by Western blot and using ImageJ to quantification. Statistical analysis was performed using one-way ANOVA followed by post hoc Tukey's tests for multiple comparisons. Shown are the means ± S.E.M values from three independent experiments. *Represents $P<0.05$, **$P<0.01$, ***$P<0.005$, and ****$P<0.001$.

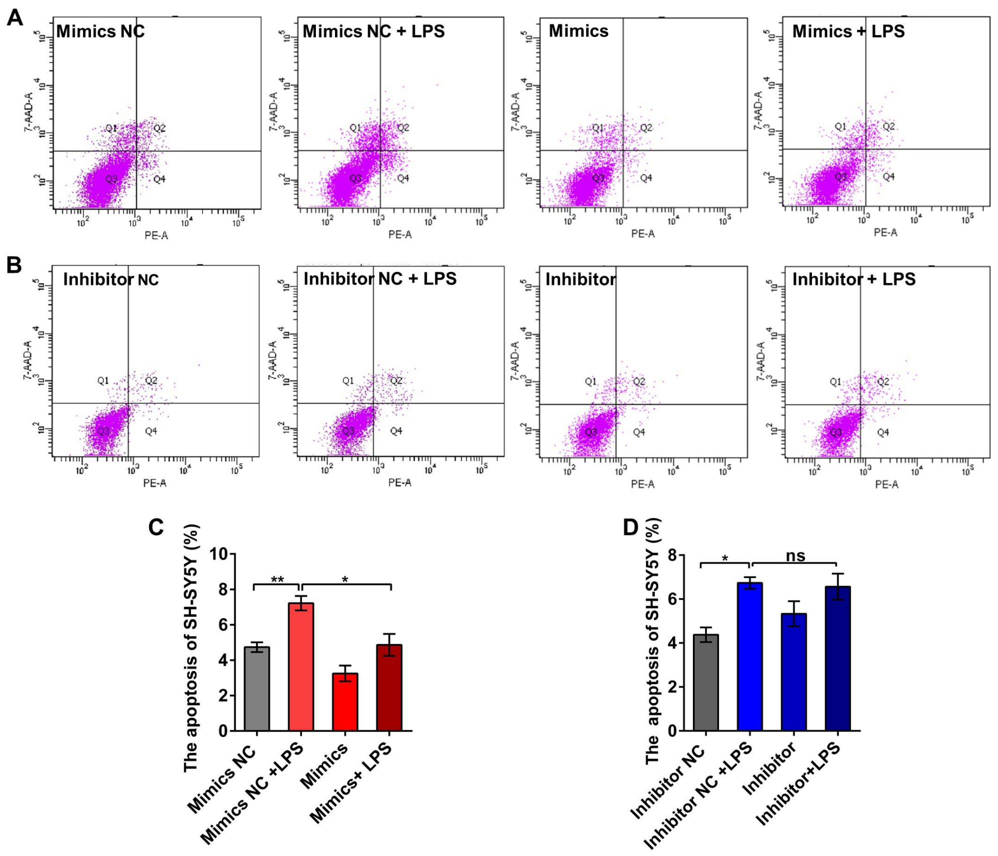

Figure S3. Increased miR-146a in Microglia prevent neurons apoptosis in LPS induced neuroinflammation. HMC3 cells were transfected with 10 nM miR-146a Mimics / 100 nM inhibitor and given 100 ng/ml LPS for 72 h, then SH-SY5Y cells were added to HMC3 cells at 1:1 ratio, and apoptosis was detected by flow cytometry after 24 h. A-B: SH-SY5Y cells and HMC3 cells were separated by flow cytometry, then go to apoptosis dectection by AnnexinV-PE/7-AAD in miR-146a Mimics (A) or inhibitor (B) group. C-D: LPS treatment of HMC3 increased the apoptosis of SH-SY5Y, an effect reduced by miR-146a mimics (C), but not in the presence of miR-146a inhibitor (D). Statistical analysis was performed using one-way ANOVA followed by post hoc Tukey's tests for multiple comparisons. Data are the means ± S.E.M values from three independent experiments. *Represents P＜0.05, **P＜0.01, ***P＜0.005, and ****P＜0.001.

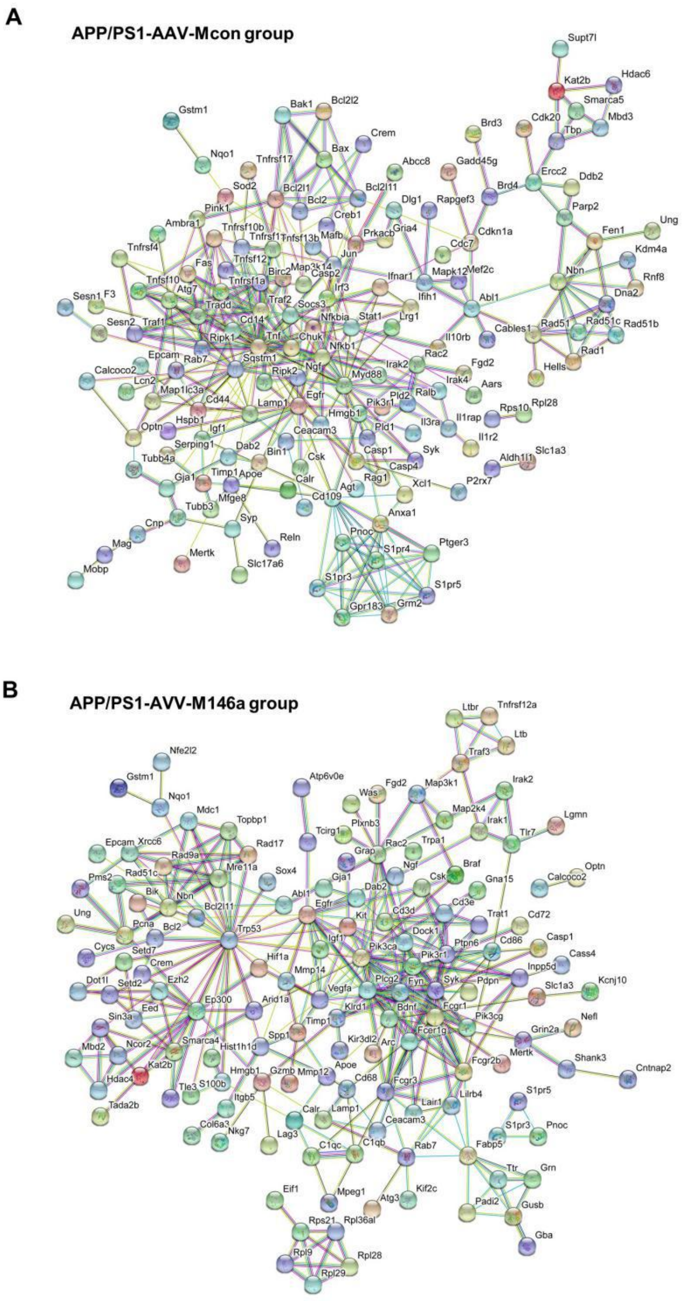

Figure S4. Network of protein interactions analysis of the microglial miR-146a overexpressed AD mouse model. A-B: Networks visualizing the functional protein association for gene signatures in APP/PS1-AAV-Mcon mice and APP/PS1-AAV-M146a mice. Nodes in the network represent proteins and edges represent protein–protein interactions, which indicate known or predicted interactions.

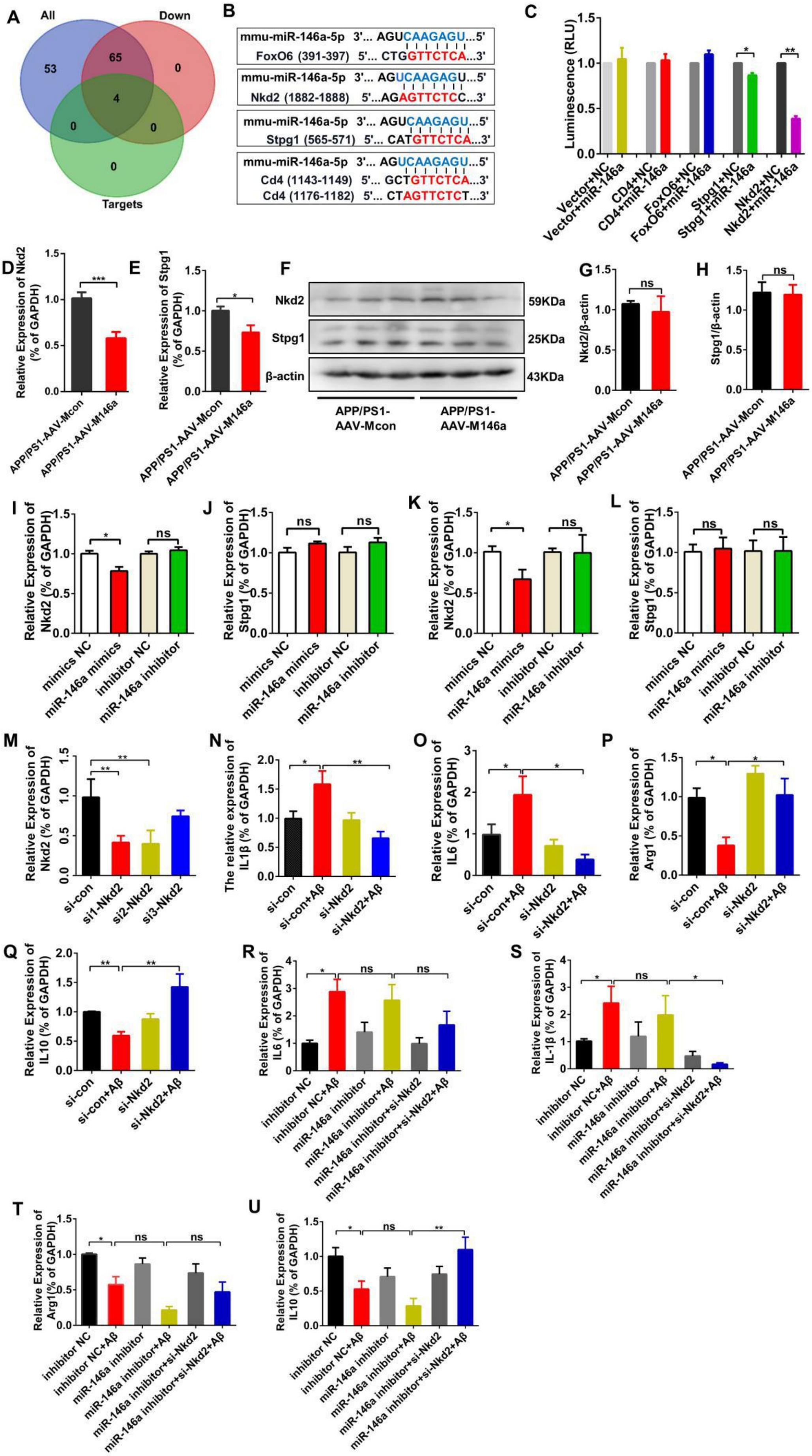

A
All
Down
Targets
53
65
0
4
0
0
0
B
mmu-miR-146a-5p 3'... AGUCAAGAGU...5'
FoxO6 (391-397) 5'... CTGGTTCTCA...3'
mmu-miR-146a-5p 3'... AGUCAAGAGU...5'
Nkd2 (1882-1888) 5'...AGAGTTCTCC...3'
mmu-miR-146a-5p 3'... AGUCAAGAGU...5'
Stpg1 (565-571) 5'... CATGTTCTCA...3'
mmu-miR-146a-5p 3'... AGUCAAGAGU...5'
Cd4 (1143-1149) 5'... GCTGTTCTCA...3'
Cd4 (1176-1182) 5'... CTAGTTCTCT...3'
C
Luminescence (RLU)
Vector+NC
Vector+miR-146a
CD4+NC
CD4+miR-146a
FoxO6+NC
FoxO6+miR-146a
Stpg1+NC
Stpg1+miR-146a
Nkd2+NC
Nkd2+miR-146a
D
Relative Expression of Nkd2 (% of GAPDH)
APP/PS1-AAV-Mcon
APP/PS1-AAV-M146a
E
Relative Expression of Stpg1 (% of GAPDH)
F
Nkd2
Stpg1
β-actin
59KDa
25KDa
43KDa
APP/PS1-AAV-Mcon
APP/PS1-AAV-M146a
G
Nkd2/β-actin
H
Stpg1/β-actin
I
Relative Expression of Nkd2 (% of GAPDH)
mimics NC
miR-146a mimics
inhibitor NC
miR-146a inhibitor
J
Relative Expression of Stpg1 (% of GAPDH)
K
L
M
si-con
si1-Nkd2
si2-Nkd2
si3-Nkd2
N
The relative expression of IL1β (% of GAPDH)
si-con+Aβ
si-Nkd2
si-Nkd2+Aβ
O
Relative Expression of IL6 (% of GAPDH)
P
Relative Expression of Arg1 (% of GAPDH)
Q
Relative Expression of IL10 (% of GAPDH)
R
inhibitor NC
inhibitor NC+Aβ
miR-146a inhibitor
miR-146a inhibitor+Aβ
miR-146a inhibitor+si-Nkd2
miR-146a inhibitor+si-Nkd2+Aβ
S
Relative Expression of IL-1β (% of GAPDH)
T
Relative Expression of Arg1 (% of GAPDH)
U
Relative Expression of IL10 (% of GAPDH)

Figure S5. Identification and validation of miR-146a target genes. A: Venn diagram of all differential genes (All), downregulating genes (Down), and target genes (Targets) were drew on the website http://bioinformatics.psb.ugent.be/webtools/Venn/. B-C: Luciferase reporter assays were used to detect the interaction between the putative FoxO6, Nkd2, Stpg1 and Cd4 binding sites with miR-146a. The wild-type (wt) FoxO6, Nkd2, Stpg1 and Cd4 3'UTRs constructs were co-transfected with miR-146a mimic in 293T cells and the relative activity of luciferase is presented as Renilla/firefly luminescence units (RLUs). Two-tailed unpaired t test with Welch's correction used for data analysis. D-E: The mRNA levels of Nkd2 and Stpg1 in APP/PS1-AAV-Mcon group and APP/PS1-AAV-M146a group were detected by qRT-PCR. F: The protein level of Nkd2 and Stpg1 were measured by Western blot. G-H: Quantification of Nkd2 and Stpg1 protein levels were measured by ImageJ in APP/PS1-AAV-Mcon group and APP/PS1-AAV-M146a group. Statistical analysis was performed using two-tailed unpaired t test with Welch's correction. I-J: 293T cells were transfected with miR-146a mimics, miRNA NC, inhibitor NC or inhibitor for 24 h, and the mRNA levels of Nkd2 and Stpg1 were detected by qRT-PCR. K-L: HMC3 cells were transfected with miR-146a mimics, miRNA NC, inhibitor NC or inhibitor for 24 h, and the mRNA levels of Nkd2 and Stpg1 were detected by qRT-PCR. Statistical analysis was performed using two-tailed unpaired t test with Welch's correction. M: HMC3 cells were transfected with si-control, Nkd2 siRNA1, siRNA2 or siRNA3 for 24 h, and the mRNA levels of Nkd2 was detected by qRT-PCR. N-Q: HMC3 cells were transfected with si-control or Nkd2 siRNA2 and given Aβ stimulate for 12 h. The mRNA level of IL-1β, IL-6, Arg1 and IL-10 were detected by qRT-PCR. R-U: HMC3 cells were transfected with miR-146a inhibitor NC, inhibitor or Nkd2 siRNA2 and given Aβ stimulate for 12 h. The mRNA level of IL-1β, IL-6, Arg1 and IL-10 were detected by qRT-PCR. Statistical analysis was performed using one-way ANOVA followed by post hoc Tukey's tests for multiple comparisons. All data are presented as the means ± S.E.M. At least three independent experiments were performed. *Representsp < 0.05, **p < 0.01, ***p < 0.001, and ****P<0.001.

Table S1 primers sequences

Primer name	Upstream primer (5′ → 3′)	Downstream primer (5′ → 3′)
**U6**	ATTGGAACGATACAGAGAAGATT	GGAACGCTTCACGAATTTG
**MiR-146a**	CCTGAGAAGTGAATTCCATGGG	TGGTGTCGTGGAGTCG
**Homo-GAPDH**	GAAGGGCTCATGACCACAGTCCAT	TCATTGTCGTACCAGGAAATGAGCTT
**Homo-IL-6**	CCTTCCAAAGATGGCTGAAA	TGGCTTGTTCCTCACTACT
**Homo-IL-1β**	CATGGGATAACGAGGCTTATG	CCACTTGTTGCTCCATATCC
**Homo-TNF-α**	GAGCCAGCTCCCTCTATTTA	GGGAACAGCCTATTGTTCAG
**Homo-Arg1**	GGGCTACTCTCAGGATTAGAT	CGAAACAAGCCAAGGTTATTG
**Homo-TGF-β**	CAGTCACCATAGCAACACTC	CCTGGCCTGAACTACTATCT
**Mus-NLRP**	ACCAGCCAGAGTGGAATGAC	ATGGAGATGCGGGAGAGATA
**Mus-Caspase1**	GCTTTCTGCTCTTCAACACC	AAAATGTCCTCCAAGTCACAAG
**Mus-ASC**	GCCAGAACAGGACACTTTGTG	AGTCAGCACACTGCCATGC
**Mus-IL-6**	TAGTCCTTCCTACCCCAATTTCC	TTGGTCCTTAGCCACTCCTTC
**Mus-IL-1β**	GGGCCTCAAAGGAAAGAATCT	GAGGTGCTGATGTACCAGTTGG
**Mus-TNF-α**	GACGTGGAACTGGCAGAAGAG	TTGGTGGTTTGTGAGTGTGAG
**Mus-Arg1**	CTTGGCTTGCTTCGGAACTC	GGAGAAGGCGTTTGCTTAGTTC
**Mus-TGF-β**	CAGAGCTGCGCTTGCAGAG	GTCAGCAGCCGGTTACCAAG
**Homo-Stpg1**	ATCTCCCAGCAGGCTTTGAC	CTCTGGAGAGGCCACAGTTG
**Homo-Nkd2**	CATGAAGTGAGAGGGGACAGG	TCTTTCTAGCACTGCTGCCTT
**Mus-GAPDH**	AAGAGGGATGCTGCCCTTAC	TACGGCCAAATCCGTTCACA
**Mus-Nkd2**	AGAGGAGAGAGAGTCCCGAAG	CTTGTCCCGTGAGCGATGTT
**Mus-CD16**	CCAGC TACACGTTTAAGGCC	TAGCGTGATGGTTTCCCCTT
**Mus-CD206**	GTGGGGACCTGGCAAGTATC	CACTGGGGTTCCATCACTCC
**Mus-IL10**	TGGTAGAAGTGATGCCCCAG	ACTCTTCACCTGCTCCACTG
**Homo-IL-10**	GTTGCCAAGCCTTGTCTGAG	CATTCTTCACCTGCTCCACG
**Mus-MFG-E8**	CGGGCCAAGACAATGACATC	TCTCTCAGTCTCATTGCACACAAG

Table S3 Homo-Nkd2 siRNA target sequences

Name	target sequences
**Homo-Nkd2 siRNA1**	CGTTCACGCTCTATGACTT
**Homo-Nkd2 siRNA2**	AGAACACGGAGCGCAGAAA
**Homo-Nkd2 siRNA3**	GCCTCATGCACACCATCTA
